# Variability and trait specific accessions for enhanced agronomic performance and nutritional traits in barnyard millet germplasm evaluated in diverse agro-ecologies in India

**DOI:** 10.3389/fpls.2026.1760632

**Published:** 2026-03-25

**Authors:** Nakka Amulya, Rumana Khan, Dinesh C. Joshi, Ramesh Singh Pal, Sharwan Kumar Shukla, Maneet Rana, Udit Prakash, Silpa Sahoo, Shweta, Ravikiran K. T., Manish Kumar Kesarwani, P. Sanjana Reddy, Ramwant Gupta, P. K. Singh, Pooran Chand, Kamaluddin, Shiv Nath, Rinni Singh, Sanjay Singh, Sushil Kumar Chaturvedi

**Affiliations:** 1 Rani Lakshmi Bai Central Agricultural University, Jhansi, Uttar Pradesh, India; 2 Indian Council of Agricultural Research (ICAR)- Vivekananda Parvatiya Krishi Anusandhan Sansthan, Almora, Uttarakhand, India; 3 Indian Council of Agricultural Research (ICAR)-Indian Grassland & Fodder Research Institute, Jhansi, Uttar Pradesh, India; 4 Chandra Shekhar Azad University of Agriculture & Technology (CSAUAT), Kanpur, Uttar Pradesh, India; 5 Indian Council of Agricultural Research (ICAR)-Central Soil Salinity Research Institute (CSSRI), Regional Research Station, Lucknow, Uttar Pradesh, India; 6 Sam Higginbottom University of Agriculture, Technology and Sciences (SHUATS), Prayagraj, Uttar Pradesh, India; 7 Indian Council of Agricultural Research (ICAR) – Indian Institute of Millets Research (IIMR), Hyderabad, India; 8 Deen DayalUpadhyaya Gorakhpur University (DDU), Gorakhpur, Uttar Pradesh, India; 9 Banaras Hindu University (BHU), Varanasi, Uttar Pradesh, India; 10 Sardar Vallabhbhai Patel University of Agriculture and Technology (SVPUAT), Meerut, Uttar Pradesh, India; 11 Banda University of Agriculture and Technology (BUAT), Banda, Uttar Pradesh, India; 12 Acharya Narendra Deva University of Agriculture and Technology (ANDUAT), Kumarganj, Ayodhya, Uttar Pradesh, India; 13 Uttar Pradesh Council of Agricultural Research, Lucknow, Uttar Pradesh, India

**Keywords:** climate resilient varieties, correleation, immune, inter cluster distance, metabolic regulation, nutri-dense, principal components

## Abstract

Millets are a diverse group of small-seeded grasses recognized for their nutritional value and adaptability to marginal environments. The present investigation assessed 39 barnyard millet (*Echinochloa* spp.) genotypes for agro-morphological and nutritional traits across two contrasting agro-ecological locations, Jhansi and Almora, during the *kharif* season of 2024. Analysis of variance revealed significant differences among genotypes for all traits studied, indicating substantial genetic variability. Correlation analysis demonstrated significant associations between grain yield and its component traits at both locations as well as under pooled analysis. In the pooled dataset, plant height (r = 0.60***) and flag leaf length (r = 0.67***) showed significant positive correlations with grain yield, highlighting their importance as yield-contributing traits. Principal component analysis generated 19 components, of which the first eight explained 78.45% of the total variation. PC1 accounted for the most variability in genetic divergence (16.70%), followed by PC2 and PC3, which contributed 13.12% and 11.82% of the total variation, respectively. Cluster analysis classified the genotypes into four distinct clusters, with Cluster III comprising the largest number of genotypes (16). The maximum inter-cluster distance (6.51) observed between Clusters I and II reflected high genetic divergence, indicating their suitability for use in hybridization programs targeting nutri-dense and climate-resilient cultivars. Cluster I emerged as the most promising group, combining superior yield and nutritional attributes. Genotypes originating from Cameroon, Russia, and India exhibited the highest grain yield, whereas accessions from Cameroon, Malawi, and Syria were notable for enhanced nutritional quality. These nutritionally superior genotypes hold potential for the development of millet-based functional foods contributing to improved digestive health, immune response, and metabolic regulation.

## Introduction

Barnyard millet (*Echinochloa* spp.) is a warm, temperate climate crop cultivated across the world, with a significant production in Asia, with India, China, Japan, and Korea among the highest (Renganathan et al., 2020). In India, it is majorly cultivated in Tamil Nadu, Andhra Pradesh, Karnataka and Uttarakhand (Gomashe, 2017). It is self-pollinated crop belonging to family *Poaceae*. The two significant and commonly cultivated species in the *Echinochloa* genus are the Japanese barnyard millet (*E. esculenta*) and the Indian barnyard millet (*E. frumentacea*) (Bhinda et al., 2023). In India, barnyard millet is predominantly cultivated during the *kharif* season and is widely distributed from the Himalayan region in the north to the Deccan Plateau in the south, thriving at elevations of up to 2300 m above mean sea level (MSL), particularly in the hilly tracts of Uttarakhand and Tamil Nadu (Singh et al., 2022; Gomashe, 2017). The crop is commonly grown on steep slopes and undulating terrains in hilly, tribal, and marginal regions, where limited crop diversification options and adverse agro-ecological conditions constrain the cultivation of other cereals (Sood et al., 2015).

It is an underutilized yet nutrient-dense minor millet (Kumar et al., 2020) having a low carbohydrate content and digest’s slowly, making it a natural gift for modern humans with sedentary lifestyle. It has potential to be included as a perfect diet in these days of rising diabetes rates. The overall nutritional composition includes; protein (10.5%), fat (3.6%), carbohydrate (68.8%), crude fiber (9.8 g/100g), ash content (4.0-4.5 g/100g) and energy (398 kcal/100 g) (Ugare et al., 2014). The total dietary fiber level was high (12.6%), including soluble (4.2%) and insoluble (8.4%) fractions (Ugare et al., 2014; Gomashe, 2017). Micronutrient content consists of iron (15-19.5 mg/100 g), zinc (2.6-4.75 mg/100 g), calcium (11-27.1 mg/100 g) and magnesium (1.33-3.13mg/100g) (Singh et al., 2022). Despite of these nutritional benefits, many antinutritional substances, particularly polyphenols, tannins, phytic and phytate acid, goitrogens, and oxalic acid, are also present in millets. The bioavailability of minerals is influenced by the presence of antinutrients such tannins, oxalates, polyphenols, and phytate. These antinutrient factors combine with dietary minerals including calcium, zinc, and iron to create complexes that significantly lower their bioavailability and render them inaccessible to human bodies (Sood et al., 2015).

Its exceptional climatic resilience enables the crop to thrive in challenging and fragile environments with low agricultural input (Maithani et al., 2023). It is ideal in an era of climate change and progressively declining natural resources since they can thrive in the most hostile environments where no other crop can survive. However, barnyard millet displays substantial variation in performance across environments due to differences in temperature, rainfall, altitude, and soil conditions, emphasizing the importance of region-specific study to identify genotypes with stable yields and superior nutritional traits. The region of Bundelkhand is situated in the central part of India, under the Indo-Gangetic plain to the north, while the rolling Vindhyan mountain range stretches across the northwest to the south. This region extends over fourteen districts *i.e.*, seven districts in Madhya Pradesh (Datia, Niwari, Tikamgarh, Chattarpur, Damoh, Sagar, and Panna) and seven districts in Uttar Pradesh (Jhansi, Jalaun, Lalitpur, Hamirpur, Mahoba, Banda, and Chitrakut) (Gupta et al., 2014). Due to its geographic location, the region is vulnerable to drought and experiences irregular, low rainfall. This has made agriculture heavily dependent on monsoons, often leading to crop failures and hardships for farmers (Prakash et al., 1998). As a disadvantaged area, Bundelkhand has limited irrigation, a large amount of wasteland, lower rainfall, and poorer soil quality compared to the state averages in Uttar Pradesh and Madhya Pradesh (Anuja, 2018; Gupta et al., 2012). Millets are traditionally grown by farming communities in Bundelkhand region (Sah et al., 2021) but green revolution leads t o a decline in area of millets in the region. Likewise, Uttarakhand is divided into two divisions (or commissionaires), Garhwal and Kumaon, which consists of 13 districts. Chamoli, Dehra Dun, Haridwar, Pauri-Garhwal, Ruder Prayag, Tehri-Garhwal, and Uttar Kashi are the seven districts that make up the Garhwal Division. Kumaon Division is divided into six districts: Almora, Bageshwar, Champawat, Nainital, Pithoragarh, and Udham Singh Nagar (US Nagar) (Sharma and Bhatkoti, 2025). Millets are also the traditional crops of Uttarakhand’ hilly landscapes. While millets are cultivated across the 8 states of India, Uttarakhand state ranks 3rd in aspect of the area occupied under millet cultivation. Uttarakhand has 48% area under cultivation of millets in comparison to other Himalayan states. Barnyard Millet (*Echinochloa crusgalli*) and Finger Millet (*Eleusine coracana*) are two prominent millets in Uttarakhand which have the value chains dominated by smallholder farmers (Bhatt et al., 2023).

Despite being an essential crop for food and nutritional security, barnyard millet’s genetic improvement has not advanced much (Gomashe, 2017). Barnyard millet is inherently climate resilient, but there are still no nutrient-dense, region-specific varieties. There is a significant amount of untapped potential for genetic enhancement and biofortification, as evidenced by the significant genetic variability found in agro-morphological and nutritional traits among various genotypes. Systematic characterization of this variability is therefore essential to bridge existing knowledge gaps and to generate a robust foundation for targeted breeding.

Accordingly, the present study was undertaken at two potential millets growing regions of India i.e. Jhansi and Almora to evaluate barnyard millet adaptability across contrasting environments. Jhansi represents the semi-arid Bundelkhand plains, while Almora represents the mid-hill Himalayan ecosystem, together providing an ideal experimental framework to assess genotype adaptability, stability, and nutritional performance. It will help to identify region-specific genotypes with stable growth, yield, and superior nutritional quality, thereby supporting the development of climate-resilient and nutritionally rich varieties for sustainable food systems and nutritional security in vulnerable regions.

## Materials and methods

### Experimental site

The experiment was conducted at two locations, i.e., Jhansi and Almora, during *Kharif* 2024. These two locations were selected as study sites because they represent two contrasting agroecological zones. The field experiment was carried out at the seed and research farm, Rani Lakshmi Bai Central Agricultural University, Jhansi (25.4488° N latitude, 78.5698° E longitude), Uttar Pradesh, and another trial was conducted at the experimental farm of ICAR-Vivekananda Institute of Hill Agriculture, located in Almora (25.35° N latitude and 79.39° E longitude). The experimental site at Jhansi recorded average maximum and minimum temperatures of 36.2 °C and 26.2 °C, respectively, during *kharif* 2024. There was also 876.4 mm of total rainfall, indicating a warm agroclimatic environment with moderate monsoon rainfall. In contrast, the Almora location experienced a cool to moderate hilly environment that was influenced by monsoonal rainfall patterns, with lower average maximum and minimum temperatures of 30.1°C and 17.5°C, respectively, and a total rainfall of 739.25 mm (**Supplementary Table 1**). These locations provide a comprehensive environmental gradient for evaluating genotype-environment interactions and developing region-specific and nutri-dense varieties.

With respect to soil properties, the soils of Jhansi are mostly neutral to strongly alkaline in nature. The SOC ranged from low to medium category in all the districts. Soil fertility attributes indicated that soils are low in available nitrogen, phosphorus and medium in available potassium. Availability of micronutrients viz. Cu and Zn were in sufficient in range and B, Fe, and Mn were in low category in Jhansi, respectively. It is clearly indicating the need of effective and proper nutrient management for better crop production and the sustainability of soil nutrient supply in this region (Prasad et al., 2020). Likewise soils of Almora district are mainly coarse textured soils rich in organic C with widely varying soil pH which ranged from very acidic to moderately alkaline in reaction. Based on the available nutrient indices (N.I.), the soils of Almora district were low in N, medium in S and B and high in rest other nutrients (Arya et al., 2019).

### Experimental material and design

Thirty-nine barnyard millet genotypes, including 36 potential barnyard millet germplasm and three checks (DHBM-93-3, CO KV-2, and VL Madira 207) were used in the present investigation. These germplasm lines belong to diverse origin and consist of genotypes from India, Japan, Russia, Syria, Pakistan, Cameroon, Malawi and unknown origin (**Supplementary Table 2**). The experiment was conducted in a randomized block design (RBD), with 3 replications. Each accession was planted in a three-row plot of 3 m length, with a row-to-row spacing of 22.5 cm.

### Observations recorded and methodology followed

Observations were recorded for 19 agro-morphological, yield and grain quality parameters including seven agro-morphological and yield related traits i.e. days to 50% flowering, days to maturity, flag leaf length, flag leaf width, panicle length, plant height, grain yield and 12 grain quality traits viz., protein, lipid, carbohydrate, crude fiber, ash, total soluble sugars, total antioxidant activity, total polyphenol content, iron, zinc, calcium, and magnesium. Observations for flag leaf length, flag leaf width, plant height, panicle length, and grain yield were recorded on five randomly selected competitive plants per plot, while days to 50% flowering and days to maturity were assessed on a whole-plot basis. The mean values of all traits are given in **Supplementary Material Table 3**. At the 50% flowering stage, five randomly selected plants from each plot, excluding border effects, were measured for flag leaf length, flag leaf width, and panicle length using a meter scale, and the mean values were recorded in centimeters. Plant height was measured from the base of the plant to the tip of its tallest leaf with the meter scale for five randomly selected plants, and their mean is represented in centimeters. Days to maturity was recorded as the number of days from sowing until the majority of plants in a plot are fully mature. At maturity, five plants are randomly selected from each plot, eliminating border plants. The panicles of each selected plant are harvested separately and dried properly. The grains are then manually threshed and cleaned to eliminate chaff and impurities, and the total grain weight per plant is measured using a digital balance. The grain yield per plant is measured in grams per plant (g/plant), and the average value of the plants is utilized for statistical analysis. The seeds are oven-dried till they have an 8–10% grain moisture content before being stored at 4°C. Samples of dried seeds weighing around 10 g were ground and passed through a 1 mm sieve. Then the crushed samples were used for nutritional content analysis.

### Quantification of nutritional parameters

#### Protein (%)

Protein was quantified using the micro-Kjeldahl method as outlined by Kundgol et al., 2014, with slight modifications. Approximately 0.1 g of the homogenized samples was weighed in triplicate with an accuracy of 0.1 mg and subsequently placed in the digestion tubes. Approximately 10 mL of ice-cold digestion mixture was introduced into the digestion tubes and allowed to pre-digest overnight. The digestion tubes were positioned in a digestion block at 420°C for 20–30 minutes until complete digestion was achieved. Ammonium in the sample was converted to ammonia through the application of an alkali, specifically NaOH, at a volume four times that of H_2_SO_4_ utilized in the digestion mixture. A 1% boric acid solution combined with bromocresol green and methyl red was utilized for ammonia trapping and as an indicator solution. The results were expressed as nitrogen percentage, which was converted to protein content using a conversion factor of 5.95.

#### Lipid (%)

The lipid content in the barnyard millet sample was determined using the Soxhlet extraction technique as outlined in the AOAC guidelines. A 2.0 g flour sample underwent drying (AOAC Method 934.01), after which the desiccated sample was extracted using petroleum ether (BP 40°C to 60°C, AOAC Method 960.39) using a semi-automatic Soxhlet extraction apparatus (Pelican Socsplox-SCS 02AS).

#### Carbohydrate (%)

Carbohydrate content was estimated using the anthrone method. A 100 mg powdered grain sample was hydrolyzed with 5 mL of 2.5 N HCl in a boiling water bath for 3 hours, cooled, and neutralized with sodium carbonate before making the volume up to 100 mL and centrifuging. Appropriate aliquots (0.5–1.0 mL) of the supernatant were taken for analysis. Anthrone reagent (4 mL), freshly prepared in ice-cold 95% sulphuric acid, was added to each tube, and the mixture was heated for 8 minutes in a boiling water bath and cooled. The resulting green to dark-green color was measured at 630 nm. A standard curve was prepared using glucose standards (0–40 µg mL^-1^), and carbohydrate concentration in the samples was determined from the calibration plot and expressed in percentage.

#### Crude fiber (%)

The estimation of crude fiber was conducted following the procedure detailed in the AOAC Official Methods, 2002. Three grams of each fat-free flour sample from each genotype were initially digested with 1.25% H_2_SO_4_, followed by washing with distilled water and filtration. This was succeeded by a second digestion with 1.25% NaOH solution, after which the samples were again washed with distilled water and filtered. The sample residue was ignited by placing the digested samples in a muffle furnace at a temperature of 550-650°C for 4 hours until grey ash was produced. The percentage of crude fiber was determined following the ignition of the samples as per the formula provided below.


$$\text{Crude fiber}=\frac{\text{Weight loss on ignition}}{\text{Weight of flour sample}}\times 100$$

#### Ash (%)

The ash content of the samples was determined using the AOAC (2005) technique. The muffle furnace temperature was set to 600°C, and empty crucibles were heated for 1 hour before cooling in a desiccator and being weighed (W1). Two g of defatted sample was placed in the crucible, and the weight was recorded (W2). The sample was charred over a flame in a muffle furnace at 600°C for eight hours. After complete ashing, the crucibles were transported to the desiccator, cooled, and weighed (W3). This process was repeated until a consistent weight was achieved. The ash was calculated using the following formula.


$$\text{Ash}=\frac{\text{Weight of ash}}{\text{Weight of sample}}\times 100$$

$$\text{Ash}=\frac{\text{W}3-\text{W}1}{\text{W}2-\text{W}1}\times 100$$

W3−W1=Weight of ash

W2−W1=Weight of sample

#### Total soluble sugars (mg/100 mg)

The total sugar content (TSS) was assessed using the anthrone method as described by Hedge and Hofreiter (1962). 200 μL of extract was aliquoted in triplicate into a test tube and fully evaporated using a water bath maintained at 100°C. One mL of double-distilled water was added to the test tube and vortexed vigorously. Simultaneously, a blank was prepared by adding 1 mL of double-distilled water to separate tubes. To prepare the standard, D-glucose was added in amounts of 0.01, 0.02, 0.04, 0.06, 0.08, and 0.1 mg to separate tubes, with the total volume adjusted to 1 mL. Following this, 4 mL of ice-cold anthrone reagent was added to each of the three sets of test tubes. The tubes underwent incubation in a water bath at 80°C for 8 minutes. Sulphuric acid in anthrone facilitates the dehydration of carbohydrates, resulting in the formation of furfural. Anthrone subsequently reacts with furfural, resulting in the formation of a green-blue color that is quantified spectrophotometrically. Absorbance for each solution was quantified at 630 nm relative to a blank control. Total soluble sugars were expressed as mg/100 mg.

### Determination of antioxidant activities

#### Sample preparation

To prepare the extract for total ployphenol and antioxidant activity determination, fine samples (1.0 g) were extracted by stirring with 20 ml of 85% methanol at 35°C and 150 rpm for 12 hours, followed by filtration through Whatman filter paper No. 1. The extraction was reiterated as previously described. The extracts were mixed, filtered, and diluted to 100 ml using 85% methanol. The extract solution, maintained in amber bottles at 4°C, functioned as the working solution (10 mg/ml) for the assessment of total phenolics and antioxidant activities. The estimation of various antioxidant activities and bioactive compounds was conducted in triplicate.

#### Determination of total polyphenol content (mg GAE/100g)

Colorimetric assays following Singleton and Rossi (1965) with slight modifications were used to measure total polyphenol content (TPC). One ml of extract (10 mg/ml) was mixed with Folin and Ciocalteu’s phenol reagent. Subsequently, 1.0 ml of saturated sodium carbonate solution was added after 3 min, and the volume was adjusted to 10 ml with distilled water. The reaction was darkened for 90 min and measured at 725 nm (Thermo Scientific UV 2600 spectrophotometer). Gallic acid was used to generate the standard curve (1-80 µg/ml). It was expressed as mg of gallic acid equivalent (GAE)/100 g extract.

#### Determination of total antioxidant activity (mM trolox equivalent/g dw)

The total antioxidant activity (TAA) of barnyard millet extracts was assessed using the phosphomolybdenum technique developed by Prieto et al., 1999; Arnao et al., 2001; Brand Williams et al., 1995. The procedure relies on the reduction of Mo (VI) to Mo (V) by the sample analyte and the subsequent production of distinct green phosphate/Mo (V) complexes. A 0.3 ml aliquot of extract solution was mixed with 2.7 ml of reagent solution (0.6 M sulphuric acid, 28 mM sodium phosphate, and 4 mM ammonium molybdate), capped, and incubated in a boiling water bath at 95°C for 90 minutes. Samples were left to equilibrate at ambient temperature, and absorbance was quantified at 695 nm. For the blank, 0.3 ml of methanol/double distilled water was combined with 2.7 ml of the reagent. A standard curve of trolox (10-100 μM) was established, and total antioxidant activity was quantified as mM trolox equivalent.

#### Micronutrient content (Fe = Iron (mg/1000g), Zn = Zinc (mg/1000g), Ca = Calcium (mg/100g), Mg = Magnesium (mg/100g))

Micronutrient profiling was conducted on whole grains as per Jackson (1969). At maturity, panicles from representative plants in each plot were harvested, threshed with a wooden mallet, and subsequently cleaned to ensure the meticulous avoidance of dust or metal contamination in the seed samples. Ground samples of 100 mg were digested in triplicate using a di-acid mixture of HClO_4_ and HNO_3_. The digested extract was adjusted to a final volume of 50 mL using a volumetric flask and subsequently filtered through Whatman No. 42 paper. The samples were analyzed using an inductively coupled plasma optical emission spectrometer (ICP-OES; model 5110, Agilent Technologies, Santa Clara, CA, USA), calibrated with standard solutions.

### Statistical analysis

Analysis of variance (ANOVA) was conducted on pooled multi-environment data using OPSTAT to evaluate the significance of genotypic effects. Estimates of genetic variability parameters, including phenotypic variance (σ²p), genotypic variance (σ²g), phenotypic and genotypic coefficients of variation (PCV and GCV), broad-sense heritability *H*^2^(*bs*), genetic advance (GA), and genetic advance as a percentage of the mean (GAM), were obtained using the tool for biometrical analysis (TBA) package following standard biometrical procedures. Pearson’s correlation coefficients among agro-morphological and nutritional traits were computed using the *metan* package in R (version 4.4.3). Multivariate analyses, including hierarchical cluster analysis and principal component analysis (PCA), were performed using the *cluster*, *factoextra*, and *FactoMineR* packages. Graphical representation of trait distributions and relationships, including violin plots and histograms, was generated using the *ggplot2*, *dplyr*, and *tidyr* packages in R. The scale used for classification of variability paraments (Sivasubramaniam and Madhava Menon, 1973) is as follows (**Table 1**).

**Table 1:** Criteria used to classify the genotypes based on variability parameters.

Variability Parameters		
1. Genotypic coefficient of variation (GCV & phenotypic coefficient of variation (PCV))		
S. N.	Range	Value
1.	Low	< 10%
2.	Moderate	10-20%
3.	High	>20%
2. Broad sense heritability		
1.	Low	< 40%
2.	Moderate	40-60%
3.	High	60-80%
4.	Very high	> 80%
3. Genetic advance		
1.	Low	< 10%
2.	Moderate	10-20%
3.	High	>20%

## Results and discussion

### Combined ANOVA for pooled data

The analysis of variance (ANOVA) revealed highly significant differences for all agro-morphological and nutritional traits, demonstrating that the experimental material has significant genetic diversity (**Table 2**). The genotypes showed high significant variation (p<0.01) for all agro-morphological and nutritional quality traits such as days to 50% flowering, days to maturity, flag leaf length, flag leaf width, panicle length, plant height, grain yield, protein, lipid, carbohydrate, crude fiber, ash, total soluble sugars, total antioxidant activity, total polyphenol content, iron, zinc, calcium, and magnesium. Location showed significant changes (p<0.01) for days to 50% flowering, panicle length, plant height, protein, ash, total polyphenol content, iron, zinc, and magnesium, whereas, p<0.05 for days to maturity, grain yield, carbohydrate, crude fiber, and total antioxidant activity, indicating that the expression of these features was significantly impacted by environmental factors in different locations. The high significant location × genotype interaction was recorded for all traits (p < 0.01), indicating that genotype performance differed considerably between locations. This implies that genotypes responded differently to changes in location and that environmental factors have a significant impact regarding the way these characteristics are expressed. As a result, multi-location testing is crucial for precise assessment of genotype stability and adaptation as well as to select location-specific or broadly adapted genotypes for nutritional characteristics and yield. Similar findings were observed by Geethanjali et al. (2023); Mishra et al. (2022) and Anuradha et al. (2014).

**Table 2:** Pooled analysis of variance (ANOVA) for all traits.

Source of variation	DF	DTFF	DTM	FLL	FLW	PL	PH	GY	Protein	Lipid	Carbohydrate	Crude fiber	Ash	TSS	TAA	TPC	Fe	Zn	Ca	Mg
Location	1	25.40***	244.67*	138.43	0.80	1140.85**	44124.56**	9.29*	5.31**	0.06	64.99*	2.03*	2.57**	34.71	60.70*	4.96**	78.13**	47.70**	119.34	240.80**
Replication within Location	4	0.07	12.91	36.78	0.17	36.60	405.59	25.86	0.18	0.19	6.05	0.13	0.08	20.38	3.75	0.10	3.57	0.10	21.40	5.00
Genotype	38	148.85**	106.37**	24.83**	0.17**	18.50**	833.56**	43.78**	1.33**	2.55**	129.78**	1.15**	0.27**	29.37**	85.10**	2.39**	389.26**	209.47**	148.73**	340.28**
Location X Genotype	38	8.32**	70.03**	12.20**	0.26**	11.50**	332.30**	5.98**	0.70**	0.67**	29.30**	0.49**	0.17**	5.39**	12.96**	0.45**	18.22*	9.45**	16.51**	29.27**
Pooled Error	152	1.27	6.61	2.42	0.06	2.09	31.50	1.57	0.24	0.34	4.85	0.07	0.04	2.18	3.44	0.10	11.41	0.65	4.97	4.16
Total	233																			

*, ** and *** indicates significance at 5%, 1% and 0.1% level of significance respectively. DTFF, Days to 50% flowering (number of days); DTM, Days to maturity (number of days); FLL, Flag leaf length (cm); FLW, Flag leaf width (cm); PL, Panicle length (cm); PH, Plant height (cm); GY, Grain yield (g/plant); Protein (%), Lipid (%), Carbohydrate (%), Crude fiber (%), Ash (%), TSS, Total soluble sugars (mg/100mg); TAA, Total antioxidant activity (mM trolox equivalent/g dw); TPC, Total polyphenol content (mg GAE/100g); Fe, Iron (mg/1000g); Zn, Zinc (mg/1000g); Ca, Calcium (mg/100g); Mg, Magnesium (mg/100g).

### Genetic variability parameters

Estimates of genetic variability parameters such as phenotypic variance, genotypic variance, phenotypic coefficient of variation, genotypic coefficient of variation, broad sense heritability, genetic advance and genetic advance as a percentage of the mean are presented in **Table 3**. Grain yield revealed high PCV (20.78%) and moderate GCV (17.85%) with high heritability (73.78%) and low genetic advance (4.64), indicating a predominance of additive gene action and suggesting that direct selection is successful. The majority of agro-morphological traits showed moderate GCV and PCV (10–20%). Days to 50% flowering showed low PCV (9.47%) and GCV (8.99%), suggesting that this trait is under strong genetic control and consists of low variability. On the other hand, moderate to high PCV and GCV (10–20%; >20%) were reported for total polyphenol content (PCV = 21.94%, GCV = 18.11%), total antioxidant activity (PCV = 15.92%, GCV = 13.44%), grain yield (PCV = 20.78%, GCV = 17.85%) and zinc (PCV = 25.88%, GCV = 25.02%), indicating the existence of significant genetic diversity and more opportunity for selection. Across variables, broad-sense heritability estimates ranged from low (<40%) to very high (>80%). Very high heritability (>80%) was demonstrated by zinc (93.48%), days to 50% flowering (90.07%), magnesium (85.74%) and iron (83.09%). While calcium (76.43%), grain yield (73.78%), total antioxidant activity (71.32%), total polyphenol content (68.16%), carbohydrate (67.26%), and total soluble sugars (61.10%) showed high heritability (60–80%). Panicle length (27.34%), protein (33.56%), ash (32.74%), and flag leaf width (9.79%) displayed low heritability (<40%), suggesting a significant environmental impact. Protein content exhibited moderate heritability 54.20% (Choudhary et al., 2023), whereas flag leaf length showed low heritability with a broad-sense estimate of ~28.16% in foxtail millet germplasm (Dharnishkumar et al., 2023). Moderate genetic advance was recorded for plant height (17.36), iron (14.87), magnesium (14.17) and zinc (11.70), indicating the effect of additive genes. Grain yield, total antioxidant activity, and micronutrient contents are among the traits with high to very high heritability and moderate genetic advance that are primarily controlled by additive gene action and can be successfully enhanced through selection. On the other hand, characteristics with low genetic advancement and heritability are heavily impacted by environmental factors and show less improvement. Similar findings were observed by Geethanjali et al. (2023); Mishra et al. (2022) and Anuradha et al. (2014).

**Table 3:** Genetic variability parameters.

Parameter	Grand mean	Phenotypic variance (σ²p)	Genotypic variance (σ²g)	Phenotypic coefficient of variation (PCV) (%)	Genotypic coefficient of variation (GCV) (%)	Broad-sense heritability *H*^2^(*bs*) (%)	Genetic advance (GA)	Genetic advance as percentage of mean (GAM) (%)
DTFF	54.92	27.04	24.36	9.47	8.99	90.07	9.65	17.57
DTM	90.64	33.81	14.52	6.41	4.20	42.94	5.14	5.67
FLL	26.17	7.79	3.41	10.66	7.05	43.79	2.52	9.62
FLW	2.32	0.11	0.01	14.35	4.49	9.79	0.07	2.90
PL	19.31	3.66	1.00	9.91	5.18	27.34	1.08	5.58
PH	148.45	215.31	123.62	9.88	7.49	57.42	17.36	11.69
GY	14.71	9.34	6.89	20.78	17.85	73.78	4.64	31.58
Protein	6.65	0.50	0.17	10.58	6.13	33.56	0.49	7.31
Lipid	6.62	0.76	0.36	13.21	9.02	46.61	0.84	12.69
Carbohydrate	64.46	29.75	20.01	8.46	6.94	67.26	7.56	11.72
Crude fiber	6.16	0.32	0.17	9.17	6.61	52.01	0.61	9.82
Ash	2.51	0.10	0.03	12.64	7.23	32.74	0.21	8.53
TSS	23.37	7.24	4.42	11.52	9.00	61.10	3.39	14.49
TAA	27.12	18.64	13.29	15.92	13.44	71.32	6.34	23.39
TPC	3.36	0.54	0.37	21.94	18.11	68.16	1.03	30.81
Fe	50.09	75.52	62.75	17.35	15.81	83.09	14.87	29.69
Zn	23.48	36.92	34.51	25.88	25.02	93.48	11.70	49.83
Ca	29.56	30.85	23.58	18.79	16.43	76.43	8.74	29.58
Mg	63.78	64.36	55.18	12.58	11.65	85.74	14.17	22.22

DTFF, Days to 50% flowering (number of days); DTM, Days to maturity (number of days); FLL, Flag leaf length (cm); FLW, Flag leaf width (cm); PL, Panicle length (cm); PH, Plant height (cm); GY, Grain yield (g/plant); Protein (%), Lipid (%), Carbohydrate (%), Crude fiber (%), Ash (%), TSS, Total soluble sugars (mg/100mg); TAA, Total antioxidant activity (mM trolox equivalent/g dw); TPC, Total polyphenol content (mg GAE/100g); Fe, Iron (mg/1000g); Zn, Zinc (mg/1000g); Ca, Calcium (mg/100g); Mg, Magnesium (mg/100g).

### Pearson’s correlation analysis

**Figure 1:**
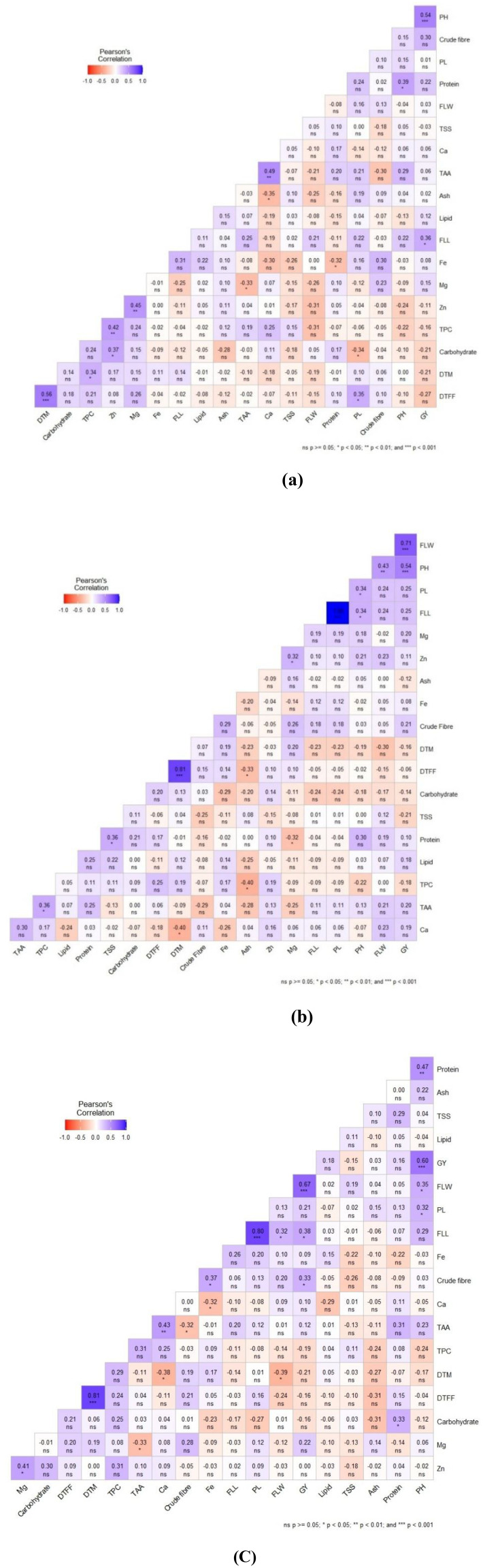
Correlation among agro-morphological and nutritional traits at **(a)** Jhansi **(b)** Almora and **(c)** pooled analysis.

Correlation analysis revealed distinct associations among agro-morphological and nutritional traits across both locations. Plant height (r = 0.54***) and flag leaf length (r = 0.36*) were significantly positively correlated with grain yield at Jhansi, suggesting the significance of these agro-morphological characteristics in enhancing yield potential. Days to maturity (r = 0.34*) and zinc (r = 0.42**) showed positive correlations with total polyphenol content, indicating a relationship between micronutrient accumulation and physiological lifespan. Contrasting correlations were observed between magnesium and zinc (r = 0.45**) and total antioxidant activity (r = −0.33*). Calcium, on the other hand, showed a significant positive correlation (r = 0.49**) with total antioxidant activity, indicating its function in boosting antioxidant capacity (**Figure 1**).

At Almora, flag leaf width (r = 0.71***) and plant height (r = 0.54***) showed a significant positive correlation with grain yield, demonstrating the significance of canopy-related features in yield enhancement. There was a positive correlation between panicle length and flag leaf length (r = 1.00***) and a positive correlation between flag leaf width and plant height (r = 0.43**), suggesting that agro-morphological features were expressed collectively. Days to maturity showed a negative correlation with calcium content (r = −0.40*) and a positive correlation with days to 50% flowering (r = 0.81***), indicating a negative correlation between calcium accumulation and maturity duration. Among nutritional characteristics, ash content was negatively correlated with days to 50% flowering (r = −0.33*) and total polyphenol content (r = −0.40*), whereas magnesium exhibited a positive association with zinc (r = 0.32*) and a negative correlation with protein content (r = −0.32*). Furthermore, there was a significant positive correlation (r = 0.36*) between total antioxidant activity (TAA) and total polyphenol content, suggesting a coordinated improvement of antioxidant characteristics (**Figure 1**).

The pooled correlation analysis for both locations showed significant correlation between yield and yield-attributing traits. Plant height (r = 0.60**) and flag leaf width (r = 0.67***) were positively correlated with grain yield, highlighting the significance of canopy and plant stature in improving yield performance across environments. Days to maturity showed a significant association with days to 50% flowering (r = 0.81***), indicating synchronized phenological development, whereas its negative correlation with calcium content (r = −0.38*) indicates a potential trade-off between calcium accumulation and crop duration. Magnesium exhibited a negative connection with total antioxidant activity (TAA) (r = −0.33**) but a significant positive correlation with zinc (r = 0.41*), suggesting that nutritional and antioxidant features exhibit distinct correlations. Additionally, TAA displayed a negative significant correlation with crude fiber (r = −0.32**) and a positive correlation with calcium (r = 0.43*), indicating coordinated and antagonistic interactions across quality-related characteristics. Overall, flag leaf width and plant height significantly positively correlate with grain yield, indicating that these characteristics may be used as reliable selection criteria for yield enhancement under pooled environmental circumstances (**Figure 1**).

### Principal component analysis

**Figure 2:**
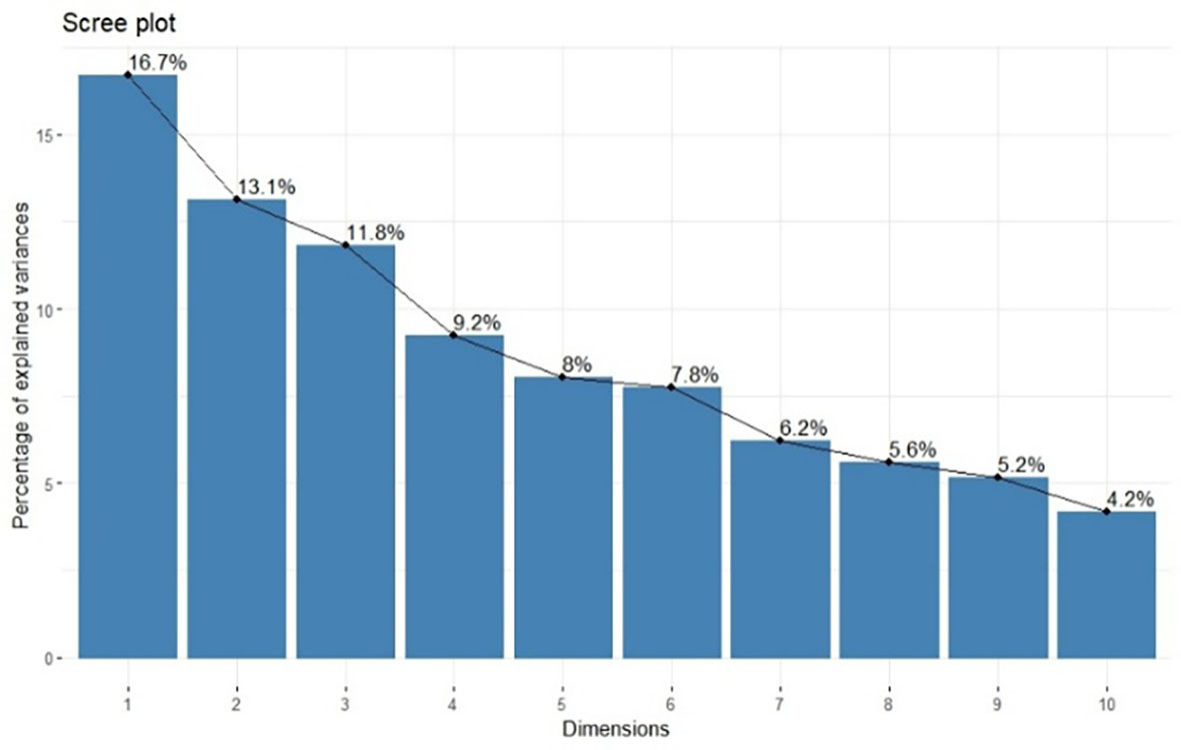
Scree plot showing Eigen values, percentage of variability of principal components.

Principal component analysis of 39 barnyard millet genotypes produced 19 principal components (**Table 4**). Eight out of 19 principal components displayed an eigenvalue greater than one. Principal component 1 had the highest eigenvalue (3.17), followed by principal components 2 and 3 (2.49 and 2.24, respectively), followed by principal component 19 (0.03), which had the lowest value. The eight principal components put together contributed to a maximum variability of 78.45% among 39 genotypes. PC1 contributed maximum variability towards genetic divergence at 16.70%. The second and third PCs contributed 13.12% and 11.82% of variation, respectively, towards total divergence (**Table 4**). The scree plot demonstrates the relative contribution of each principal component to the total variance in the dataset. The first three components account for 41.6% of the cumulative variance, indicating that major data structure can be captured with relatively few dimensions (**Figure 2**).

**Table 4:** Total variance explained by different principal components.

PC	Eigenvalue	Variance%	Cumulative variance %
1	3.17	16.70	16.70
2	2.49	13.12	29.82
3	2.24	11.82	41.63
4	1.75	9.23	50.86
5	1.53	8.03	58.89
6	1.47	7.75	66.64
7	1.18	6.21	72.85
8	1.06	5.60	78.45
9	0.98	5.17	83.61
10	0.79	4.17	87.78
11	0.64	3.39	91.17
12	0.46	2.40	93.57
13	0.36	1.87	95.44
14	0.27	1.43	96.86
15	0.20	1.07	97.93
16	0.18	0.95	98.88
17	0.10	0.53	99.41
18	0.08	0.44	99.85
19	0.03	0.15	100.00

The contribution of different agro-morphological and nutritional quality traits to the first eight principal components is presented in **Table 5**. Grain yield (17.66%), plant height (14.82%), flag leaf width (14.50%), flag leaf length (12.47%), and days to maturity (10.05%) were the key traits influencing PC1, suggesting that significant yield and yield attributing traits were the primary contributors of variation along this axis. PC2 was mostly contributed by days to maturity (15.87%), crude fiber (14.97%), days to 50% flowering (13.57%), and iron (13.23%), indicating the significance of maturity duration and nutritional characteristics in explaining variability in this component.

**Table 5:** Contribution of different traits to the first eight principal components.

Traits	PC1	PC2	PC3	PC4	PC5	PC6	PC7	PC8
DTFF	5.90	13.57	8.02	0.97	4.02	0.12	6.93	0.01
DTM	10.05	15.87	0.89	3.34	3.60	0.00	1.10	0.24
FLL	12.47	5.69	1.66	3.74	1.64	5.48	1.21	5.80
FLW	14.50	0.02	0.17	0.60	0.44	14.44	0.09	9.49
PL	8.48	8.54	1.81	1.76	0.39	18.64	0.46	5.32
PH	14.82	0.17	3.63	0.03	9.00	0.06	1.51	10.40
GY	17.66	2.13	1.01	3.29	0.03	9.72	0.02	2.54
Protein	1.39	1.64	17.42	3.28	12.93	1.32	0.47	1.72
Lipid	0.08	0.20	0.42	8.72	0.15	10.27	27.36	8.34
Carbohydrate	3.77	0.73	10.72	0.82	1.37	11.69	0.56	0.53
Crude fiber	0.37	14.97	0.36	6.54	0.25	9.26	6.00	2.58
Ash	3.12	1.61	5.16	2.27	8.82	10.87	1.47	1.35
TSS	0.06	4.71	0.14	6.79	13.83	0.60	3.99	33.16
TAA	0.96	2.43	18.05	3.24	12.05	3.36	0.92	8.24
TPC	5.15	0.09	13.35	0.00	5.09	0.00	7.21	4.87
Fe	0.44	13.23	1.59	3.44	13.07	0.43	1.20	0.00
Zn	0.47	0.62	7.80	12.67	0.12	1.57	28.47	1.27
Ca	0.16	7.36	7.71	11.10	6.90	0.89	6.50	4.12
Mg	0.14	6.40	0.08	27.39	6.30	1.28	4.53	0.00

DTFF, Days to 50% flowering (number of days); DTM, Days to maturity (number of days); FLL, Flag leaf length (cm); FLW, Flag leaf width (cm); PL, Panicle length (cm); PH, Plant height (cm); GY, Grain yield (g/plant); Protein (%), Lipid (%), Carbohydrate (%), Crude fiber (%), Ash (%), TSS, Total soluble sugars (mg/100mg); TAA, Total antioxidant activity (mM trolox equivalent/g dw); TPC, Total polyphenol content (mg GAE/100g); Fe, Iron (mg/1000g); Zn, Zinc (mg/1000g); Ca, Calcium (mg/100g); Mg, Magnesium (mg/100g).

Total antioxidant activity (18.05%), protein content (17.42%), total polyphenol content (13.35%), and carbohydrate content (10.72%) all contributed significantly to PC3, suggesting that this component mostly reflected variance related to nutritional quality parameters. PC4 was predominantly influenced by magnesium (27.39%), followed by zinc (12.67%), calcium (11.10%), and lipid content (8.72%), underscoring the significance of mineral composition in distinguishing genotypes along this axis. Total soluble sugars (13.83%), iron (13.07%), protein (12.93%), total antioxidant activity (12.05%), and plant height (9.00%) all contributed significantly to PC5, indicating the combined impact of growth-related and nutritional characteristics. The initial principal components were mainly impacted by grain yield, plant height, flag leaf length, flag leaf width, days to flowering and days to maturity, as well as essential nutritional and mineral traits including protein content, total amino acids, zinc, iron, magnesium, and total soluble sugars. These results indicate that these traits are the primary factors of genetic divergence and should be given preference in selection and improvement programs.

**Figure 3:**
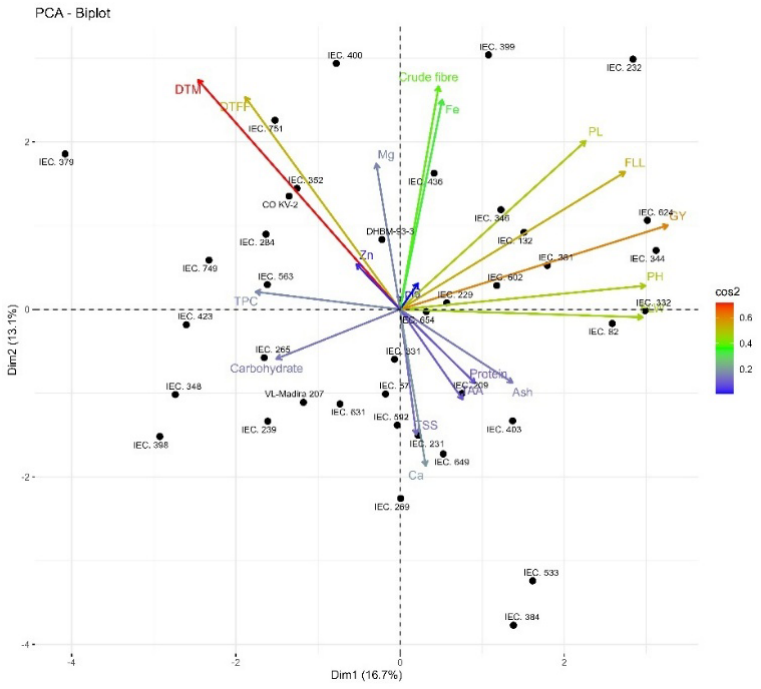
Bi-plot showing variation on the basis of score of barnyard millet genotypes with respect to traits in principle component analysis.

The PCA biplot demonstrated significant variance among genotypes for agro-morphological and nutritional parameters, with PC1 (16.7%) and PC2 (13.3%) collectively accounting for almost 30% of the total variation. PC1 was most closely associated to yield and yield attributing characteristics (plant height, flag leaf length, panicle length, and grain yield), grouping genotypes such as IEC 624, IEC 332, and IEC 82 on the positive axis, but total polyphenol content and carbohydrate exhibited negative loadings, showing an inverse correlation with yield traits. PC2 was predominantly affected by crude fiber and iron, which had positive loadings, while calcium demonstrated a significant negative correlation, differentiating genotypes according to their nutritional content. The acute angles between the plant height, flag leaf length, panicle length, and grain yield vectors indicated significant positive interrelationships, whereas obtuse angles between yield and nutritional traits suggested trade-offs between productivity and quality. Genotypes identified more distant from the origin contributed more to genetic divergence, suggesting a wide range of variability that can be used successfully for trait-specific parent selection in breeding programs (**Figure 3**). Sneha et al. (2023); Elangovan et al. (2022); Nandini et al. (2020); Arya et al. (2018) and Patil et al. (2021) all reported similar findings.

### Cluster analysis

**Figure 4:**
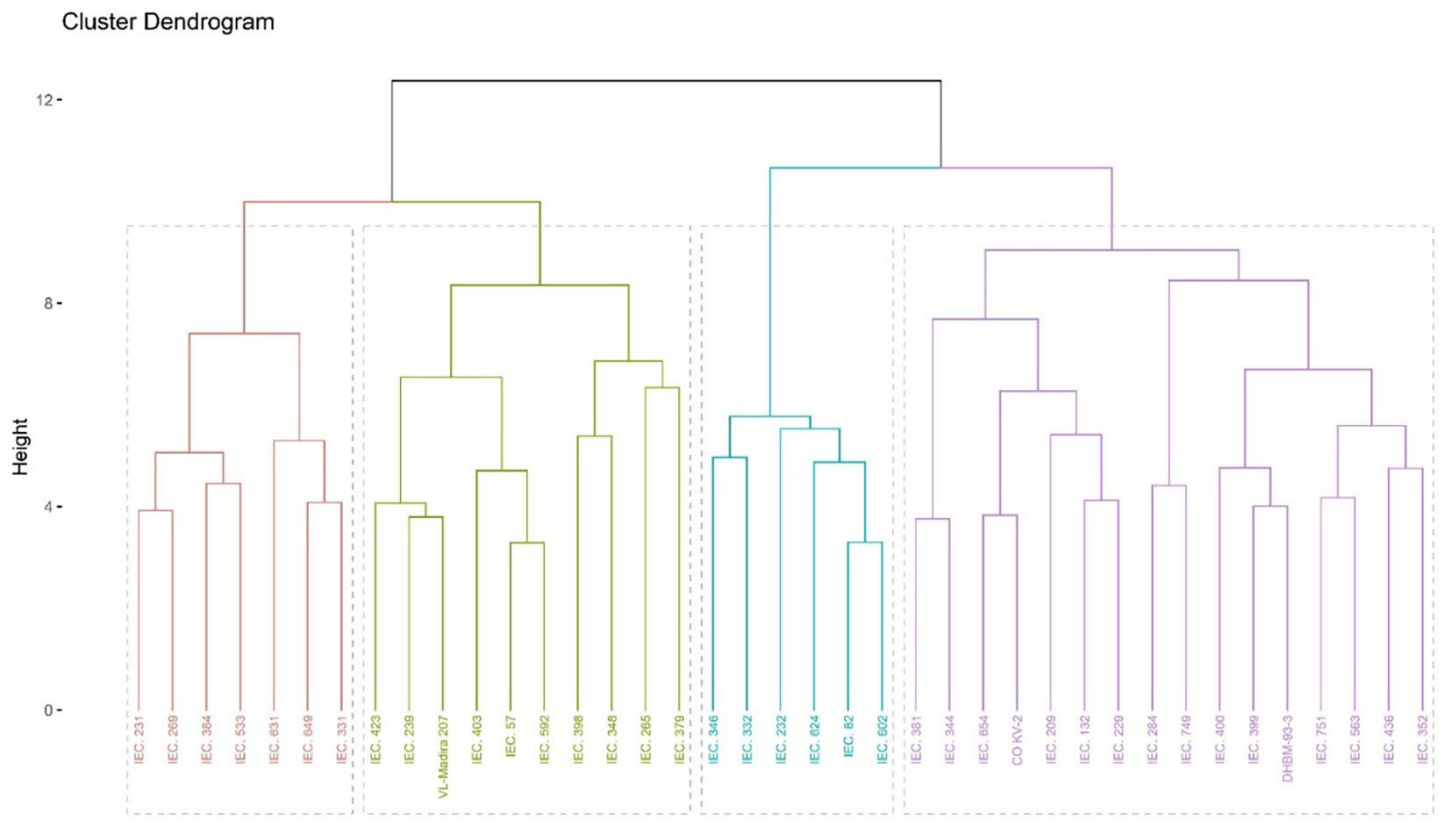
Dendrogram representing diversity in barnyard millet accessions.

Cluster analysis by Ward’s minimum variance method in barnyard millet germplasm grouped diverse accessions into four clusters (**Figure 4**). Cluster III had the highest number of genotypes, *i.e*., 16, followed by Cluster I with 10 genotypes. Cluster II and IV are represented by 6 & 7 genotypes, respectively (**Table 6**).

**Table 6:** Barnyard millet germplasm in four different clusters.

Cluster	Genotypes
Cluster I	IEC. 57, IEC. 239, IEC. 265, IEC. 379, IEC. 398, IEC. 403, IEC. 592, IEC. 348, IEC. 423, VL-Madira 207
Cluster II	IEC. 82, IEC. 232, IEC. 346, IEC. 602, IEC. 332, IEC. 624
Cluster III	IEC. 132, IEC. 209, IEC. 229, IEC. 284, IEC. 381, IEC. 399, IEC. 400, IEC. 436, IEC. 749, IEC. 751, IEC. 344, IEC. 352, IEC. 563, IEC. 654, CO KV-2, DHBM-93-3
Cluster IV	IEC. 231, IEC. 269, IEC. 384, IEC. 533, IEC. 631, IEC. 649, IEC. 331

The highest inter-cluster distance was between clusters I and II (6.51), followed by clusters I and III (6.37) (**Table 7**). Clusters I and II consist of diverse genotypes, so the hybridization between these genotypes produces desirable progenies. The highest intra cluster distance was found in cluster III (5.71), followed by cluster I (5.59).

**Table 7:** Intra and inter cluster distances between clusters.

Clusters	Cluster 1	Cluster 2	Cluster 3	Cluster 4
Cluster 1	5.59	6.51	6.37	6.07
Cluster 2		4.93	6.16	6.23
Cluster 3			5.71	6.17
Cluster 4				5.11

Cluster 1 is characterized by high levels of lipid (7.00), crude fiber (6.36), ash (2.59), magnesium (67.54), iron (59.16), and panicle length (24.17), alongside moderate grain yield (16.03), suggesting the presence of nutritionally dense and agronomically robust accessions. Cluster 2 is represented by high levels of TSS (23.77) and calcium (30.95). The elevated levels of days to maturity (92.37), carbohydrate (66.01), TPC (3.99), and zinc (25.62) are characteristics of Cluster 3. Cluster 4 is characterized by significant amounts of flag lead width (2.47), grain yield (17.05), protein (6.95), and total antioxidant activity (28.48) (**Table 8**).

**Table 8:** Cluster means of barnyard millet germplasm for all traits.

Cluster	1	2	3	4
DTFF	**55.66**	55.15	55.33	53.55
DTM	92.04	90.70	**92.37**	88.14
FLL	**27.13**	25.52	25.46	26.96
FLW	2.31	2.27	2.28	**2.47**
PL	**24.17**	22.56	22.45	23.96
PH	152.69	145.21	129.30	**163.23**
GY	16.03	13.84	11.75	**17.05**
Protein	6.53	6.75	6.10	**6.95**
Lipid	**7.00**	6.64	6.40	6.39
Carbohydrate	60.82	65.18	**66.01**	65.36
Crude fiber	**6.36**	6.00	6.23	6.23
Ash	**2.59**	2.47	2.41	2.57
TSS	22.56	**23.77**	23.30	23.41
TAA	25.85	27.50	25.77	**28.48**
TPC	3.03	3.28	**3.99**	3.35
Fe	**59.16**	43.66	56.48	49.21
Zn	23.24	22.92	**25.62**	23.26
Ca	26.29	**30.95**	29.00	30.36
Mg	**67.54**	62.34	63.38	63.27

DTFF, Days to 50% flowering (number of days); DTM, Days to maturity (number of days); FLL, Flag leaf length (cm); FLW, Flag leaf width (cm); PL, Panicle length (cm); PH, Plant height (cm); GY, Grain yield (g/plant); Protein (%), Lipid (%), Carbohydrate (%), Crude fiber (%), Ash (%); TSS, Total soluble sugars (mg/100mg); TAA, Total antioxidant activity (mM trolox equivalent/g dw); TPC, Total polyphenol content (mg GAE/100g); Fe, Iron (mg/1000g); Zn, Zinc (mg/1000g); Ca, Calcium (mg/100g); Mg, Magnesium (mg/100g).The bold values indicate highest mean values for respective traits.

According to the cluster analysis, wide genetic diversity was recorded in the barnyard millet germplasm. Cluster I and Cluster II had the greatest inter-cluster distance, suggesting that hybridization between these clusters would be most successful in producing superior progeny. Because of its higher level of agro-morphological and nutritional traits, Cluster I is identified as highly advantageous, suggesting its potential utility in breeding programmes focused on biofortification and yield improvement. Similar results were recorded by Mane et al. (2025); Patil et al. (2021); Nandini et al. (2020); Dhanalakshmi et al. (2019); Arya et al. (2018) and Selvi et al. (2015).

### Violin plot analysis

**Figure 5:**
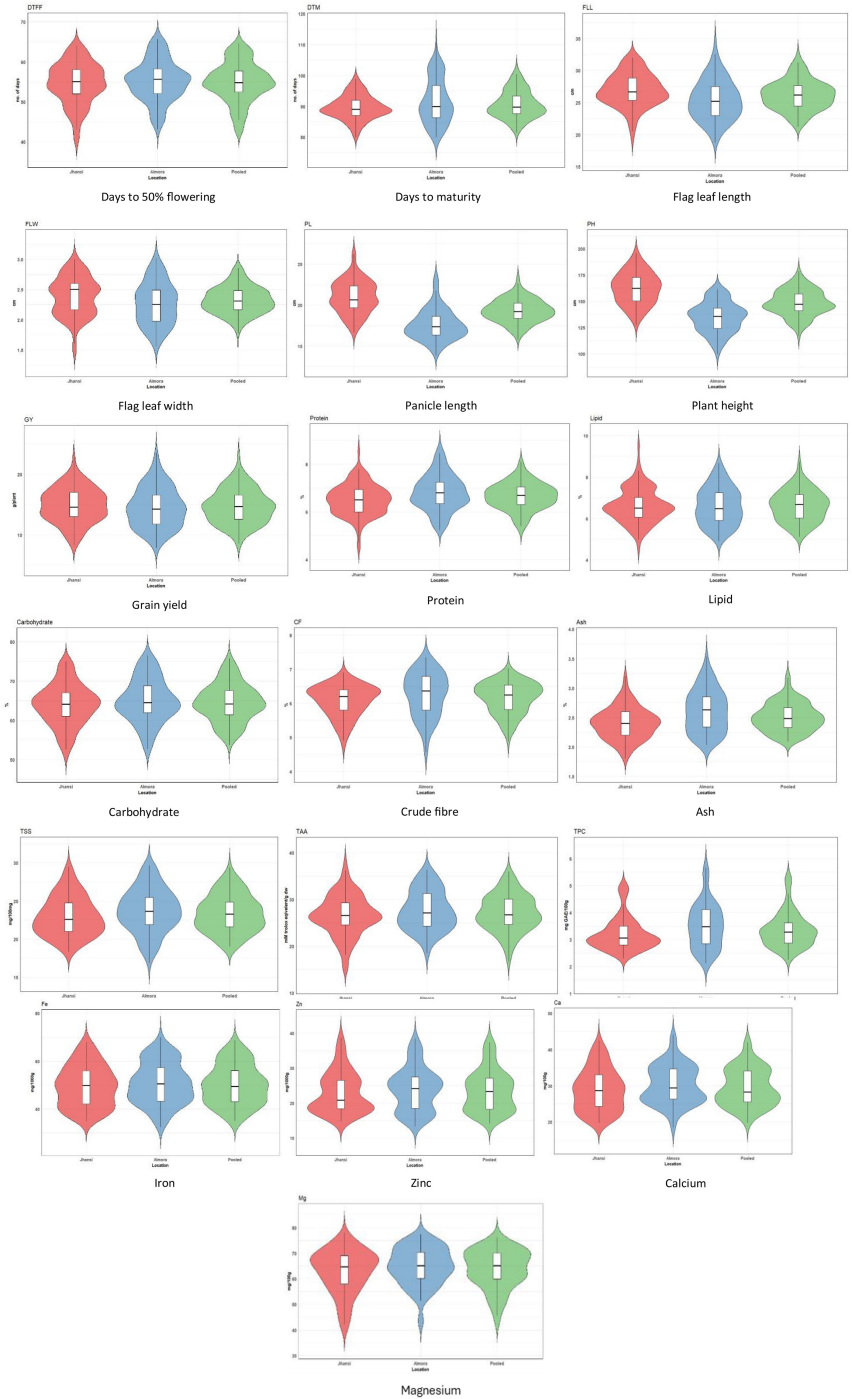
Violin plots for various traits.

Violin plots were used to visualize the distribution of data among genotypes (**Figure 5**). Each plot integrates a box plot with a kernel density estimate, allowing simultaneous representation of the central tendency, spread, and distribution shape of the data. Across both environments, the nutrient traits showed similar central tendencies but varying degrees of dispersion. Median DTFF remained stable at ~55 days in Jhansi, 56 days in Almora, and 55 days in the pooled analysis over both locations, while variability was wider at Almora (45–65 days) than Jhansi (42–64 days). Median days to maturity showed minor differences, *i.e.*, 89 days (Jhansi), 91–92 days (Almora), and 90 days (pooled), but Almora again displayed a broader range (81–104 days) compared with Jhansi (85–95 days). Flag leaf traits were largely comparable: FLL medians were 26.5 cm (Jhansi), 25 cm (Almora), and 26 cm (pooled), with wider dispersion at Almora (20–32 cm) than Jhansi (21–32 cm); FLW medians were 2.5 cm, 2.2 cm, and 2.3 cm, respectively, with greater variability at Almora (1.78–2.89 cm) than Jhansi (2.03–2.83). Panicle length showed modest shifts, with median PL of 20.5 cm (Jhansi), 18 cm (Almora), and 19.5 cm (pooled), and Almora exhibiting the broadest range (20–32 cm). Plant height differed more noticeably, with medians of 160 cm (Jhansi), 135 cm (Almora), and 145 cm (pooled), and higher variation at Jhansi (134–188 cm) than Almora (103–159 cm). Grain yield remained similar across sites with medians of 14 g/plant (Jhansi), 13 g/plant (Almora), and 14 g/plant (pooled), although Almora again showed the greatest variability (9–22 g/plant).

Protein content had median values of 6.3% (Jhansi), 6.7% (Almora), and 6.6% (pooled), with Almora showing the widest range (5.36–8.14%). Lipid content medians were 6.4%, 6.2%, and 6.5%, respectively, again with greater variability at Almora (5.4–8.2%). Carbohydrate content remained comparable at 64% (Jhansi), 65% (Almora), and 64% (pooled), with Almora displaying the broadest spread (55–76%). Crude fiber medians were 6.2%, 6.4%, and 6.3%, with Almora showing higher variability (4.8–7.2%) than Jhansi. Ash content showed slight differences of 2.4%, 2.6%, and 2.5% with wider variation at Almora (2.0–3.3%). TSS medians were 22.5, 23.5, and 23 mg/100 mg, with Almora again showing greater dispersion (18–29 mg/100 mg). Total antioxidant activity medians were 26.5, 26, and 27 mM trolox equivalent/g dw, with Almora presenting the widest range (20–35 mM trolox equivalent/g dw). Total polyphenol content medians were 3.0, 3.5, and 3.3 mg GAE/100 g, with Almora showing pronounced variability (2.3–5.5 mg).

Iron medians showed close agreement at 48, 50, and 49 mg/1000 g, while Almora exhibited wider ranges (33–67 mg/1000 g). Zinc showed moderate shifts with medians of 20, 24, and 23 mg/1000 g, again with maximum variability at Almora (15–38 mg/1000 g). Calcium medians were 28, 30, and 29 mg/100 g, with Almora showing broader ranges (21–43 mg/100 g), and magnesium medians were 64, 65, and 64 mg/100 g with greater variability at Almora (44–76 mg/100 g). Overall, Almora consistently exhibited wider trait distributions, suggesting greater environmental influence on nutrient expression.

### Histogram

Histograms were constructed to display the frequency distribution and variability. Across both environments, the genotypes showed stable average performance with varying degrees of spread. Days to 50% flowering remained consistent, with mean values of 55–56.days at both Jhansi and Almora. Days to maturity was more compact at Jhansi (87–94 days; mean ~90 days) compared with Almora (85–103 days; mean ~91 days), indicating greater environmental influence at Almora. Flag leaf traits were similar across locations: flag leaf length averaged 26–27 cm at Jhansi (24–30 cm) and ~27 cm at Almora (22–32 cm), while flag leaf width averaged 2.3–2.4 cm (2.1–2.7 cm) at Jhansi and 2.4–2.5 cm (1.9–3.0 cm) at Almora. Panicle length showed uniformity at Jhansi (19–23 cm; mean 22–23 cm) but wider variation at Almora (21–32 cm; mean ~24 cm). Plant height ranged between 145 and 180 cm at Jhansi (mean 155–160 cm) and 110 and 155 cm at Almora (mean 148–150 cm), indicating shorter plants and greater dispersion under Almora conditions. Grain yield was comparable across sites, averaging 15–16 g/plant at Jhansi (12–18 g/plant) and ~15 g/plant at Almora (10–22 g/plant), with Almora showing higher variability despite similar mean productivity. Because of the different environments across the sites Jhansi’s warmer, semi-arid climate may encourage heat-resilient types, whereas Almora’s milder, higher terrain generates patterns of balanced output; this variation has a noticeable site-specific impact on grain production.

**Figure 6:**
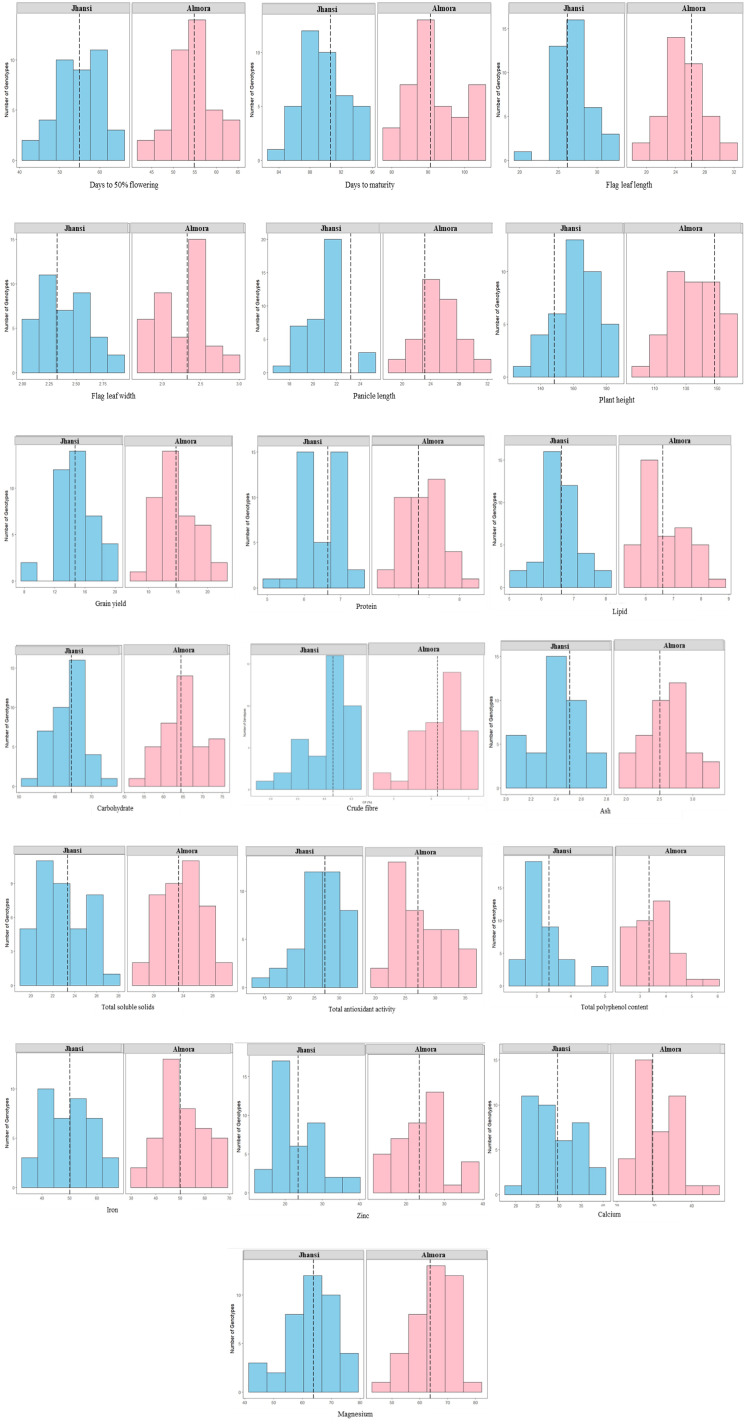
Histogram for various traits.

Protein content at Jhansi ranges from 5.8% to 7.2% (mean ≈6.6%), while at Almora it spans 5.5% to 8.3% (mean 6.8–7.0%), indicating broader variation and slightly higher values at Almora. Carbohydrates at Jhansi fall between 58% and 70% (mean 64–65%) compared to Almora’s wider 55–75% range (mean 66–67%). Lipid content at Jhansi lies within 5.5–7.5% (mean 6.5–6.7%), whereas Almora shows 5.5–8.8% (mean 6.8–7.0%). Ash content at Jhansi is narrowly distributed (2.2–2.7%, mean ≈2.5%), while Almora ranges from 2.1 to 3.1% (mean 2.6–2.7%). TSS at Jhansi varies from 21–26 mg/100 mg (mean 23–24), compared to 20–29 mg/100 mg at Almora (mean 24–25). TAA at Jhansi spans 22–32 mM TE/g dw (mean 27–28), while Almora ranges 20–36 (mean 28–29). TPC at Jhansi falls within 2.8–3.6 mg GAE/100 g (mean 3.2–3.3), whereas Almora shows 2.7–5.8 (mean 3.4–3.5), indicating greater variability and generally higher values across traits at Almora. Across locations, micronutrient contents showed similar mean levels with varying degrees of dispersion. At Jhansi, Fe, Zn, Ca, and Mg exhibited moderately narrow ranges centered around 50 mg/1000 g, 24–25 mg/1000 g, 30 mg/100 g, and 63–65 mg/100 g, respectively. Almora displayed wider distributions for all four traits, with Fe spanning 35–70 mg/1000 g, Zn 17–38 mg/1000 g, Ca 25–42 mg/100 g, and Mg 50–80 mg/100 g, although mean values remained comparable to those at Jhansi. This suggests that while environmental influence increased variability at Almora, the overall average micronutrient accumulation remained consistent across environments (**Figure 6**).

Looking upon the variability across the germplasm of different center of origin, the agro-morphological and nutritional traits of barnyard millet accessions revealed considerable variability across different geographical origins (**Table 9**). The highest mean value for days to 50% flowering (DTFF) was recorded for accessions from Pakistan (58.50 days), while the lowest was from Syria (50.92 days). For days to maturity (DTM), the highest value was observed in accessions of unknown origin (93.72 days), with the lowest in those from Syria (86.30 days). There was considerable variation among the different origins for all agro-morphological traits studied. Cameroon exhibited the highest mean values for flag leaf length (30.92 cm), plant height (165.13 cm), highest flag leaf width (2.55 cm) and grain yield (16.92 g), highlighting its potential as a genetic source for these traits. In contrast, Malawi and unknown origins recorded the lowest mean values for most traits, including the lowest flag leaf length (24.13 cm for unknown), flag leaf width (2.20 cm for unknown), panicle length (21.06 cm for unknown), plant height (131.00 cm for Malawi), and grain yield (11.39 g for Malawi). Syrian accessions exhibited the highest protein (7.14%) and lipid (7.21%) content, indicating their superior nutritional quality for these macronutrients. Cameroon showed the highest carbohydrate content (69.70%), suggesting its genotypes are potential sources for high-energy diets.

**Table 9:** Mean values for all traits across different geographical origin.

Origin	India	Japan	Russia	Syria	Pakistan	Malawi	Cameroon	Unknown
DTFF	54.98	54.74	52.12	50.92	**58.50**	55.06	57.22	55.92
DTM	90.86	90.49	87.75	86.30	92.58	93.39	86.37	**93.72**
FLL	25.95	25.48	28.46	27.25	27.09	24.56	**30.92**	24.13
FLW	2.33	2.33	2.32	2.29	2.33	2.23	**2.55**	2.20
PL	22.98	22.64	24.87	25.03	24.67	21.81	**27.06**	21.06
PH	148.28	147.54	156.35	156.36	141.81	131.00	**165.13**	144.05
GY	15.00	14.78	15.32	15.01	12.44	11.39	**16.92**	12.77
Protein	6.69	6.69	6.65	**7.14**	6.63	6.07	6.81	6.11
Lipid	6.62	6.90	6.94	**7.21**	5.93	6.30	6.28	6.18
Carbohydrate	64.93	65.15	57.65	63.62	65.21	63.14	**69.70**	64.95
Crude fiber	6.12	6.05	6.07	6.53	6.51	5.49	6.39	**6.80**
Ash	2.53	**2.58**	2.49	2.33	2.57	2.14	2.51	2.34
TSS	23.03	24.25	23.96	23.59	23.81	**27.25**	25.63	20.74
TAA	**28.37**	24.59	27.63	27.97	23.59	24.71	25.13	23.04
TPC	3.48	3.24	2.87	3.64	3.11	**3.85**	2.68	3.09
Fe	49.82	45.71	55.12	**65.72**	45.80	48.11	42.16	58.24
Zn	25.40	17.20	17.54	**34.53**	24.29	20.49	27.08	18.38
Ca	29.87	29.67	29.44	30.24	30.23	**32.33**	27.78	24.23
Mg	63.61	65.63	56.87	**71.94**	62.55	67.52	71.90	62.77

DTFF, Days to 50% flowering (number of days); DTM, Days to maturity (number of days); FLL, Flag leaf length (cm); FLW, Flag leaf width (cm); PL, Panicle length (cm); PH, Plant height (cm); GY, Grain yield (g/plant); Protein (%), Lipid (%), Carbohydrate (%), Crude fiber (%), Ash (%), TSS, Total soluble sugars (mg/100mg); TAA, Total antioxidant activity (mM trolox equivalent/g dw); TPC, Total polyphenol content (mg GAE/100g); Fe, Iron (mg/1000g); Zn, Zinc (mg/1000g); Ca, Calcium (mg/100g); Mg, Magnesium (mg/100g).The bold values indicate highest mean values for respective traits.

Crude fiber content was highest in accessions of unknown origin (6.80%), while the lowest fiber content was recorded for Malawi (5.49%). For ash content, the highest value was observed in Japanese accessions (2.58%), and the lowest in Malawi (2.14%). Malawi accessions had the highest total soluble sugars (27.25 mg/100 mg) and also showed the highest value for total polyphenol content (3.85 mg GAE/100 g). India had the highest total antioxidant activity (28.37 mM trolox equivalent/g dw), followed closely by Syria and Russia. In contrast, accessions of unknown origin were generally lowest in TSS (20.74 mg/100 mg) and TAA (23.04 mM Trolox equivalent/g dw). Cameroon had the lowest TPC (2.68 mg GAE/100 g). Syrian accessions showed the highest iron (65.72 mg/1000 g) and zinc (34.53 mg/1000 g) contents, making them especially valuable for improving these important nutrients. Cameroon and Syria also had the highest magnesium (71.90 mg/100 g and 71.94 mg/100 g, respectively), while Malawi had the highest calcium (32.33 mg/100 g). In contrast, Cameroon had the lowest iron and calcium levels among the origins evaluated.

Genotypes from Cameroon, Russia and India exhibited the highest grain yield, indicating strong yield potential under their respective growing conditions. Nutritionally, Cameroon and Malawi exhibited higher carbohydrate, protein, and mineral contents, while Syria showed the greatest iron and zinc concentrations. These findings suggest that barnyard millet accessions possess distinct nutritional and biochemical diversity influenced by their geographical origin.

## Conclusion

In the present investigation, barnyard millet accessions of diverse origin were evaluated for agro-morphological and grain quality traits. Substantial variation was observed among genotypes for protein, lipid, carbohydrate, crude fiber, ash, total soluble sugars (TSS), total antioxidant activity (TAA), and micronutrients including iron, zinc, calcium, and magnesium. Among the genotypes, IEC 232 recorded the highest grain yield (21.19 g/plant) along with high iron (58.36 mg/1000g), zinc (26.16 mg/1000g), and magnesium (65.88 mg/100g) contents. Similarly, IEC 82 exhibited high grain yield (20.11 g/plant) coupled with high protein content (7.15%) and appreciable concentrations of iron (52.09 mg/1000g), zinc (26.77 mg/1000g), and magnesium (61.57 mg/100g). IEC 209 also combined high grain yield (18.73 g/plant) with exceptionally high calcium (41.85 mg/100 g) and magnesium (74.36 mg/100g), indicating its potential for mineral-rich grain production. Although moderate in yield (15.01 g/plant), IEC 346 recorded the highest iron content (65.72 mg/1000g) along with high zinc (34.53 mg/1000g) and magnesium (71.94 mg/100g). In contrast, IEC 265 exhibited the highest zinc concentration (36.98 mg/1000g) accompanied by elevated calcium (37.20 mg/100 g) and magnesium (70.93 mg/100g), highlighting both genotypes as elite donor parents for mineral biofortification programs. Overall, the observed variability in nutritional and biochemical traits among barnyard millet accessions reflects their genetic and geographical diversity and underscores their potential use in breeding programs aimed at enhancing grain yield and micronutrient content.

## Data Availability

The original contributions presented in the study are included in the article/**Supplementary Material**, further inquiries can be directed to the corresponding author/s.

## Supplementary material

Supplementary Table 1 Meteorological data recorded during *Kharif* 2024.

Supplementary Table 2 List of barnyard millet genotypes used in the study with their origin.
