# Supplementary material for: Variability and trait specific accessions for enhanced agronomic performance and nutritional traits in barnyard millet germplasm evaluated in diverse agro-ecologies in India
Source: Front Plant Sci. 2026 Mar 25;17:1760632. doi: 10.3389/fpls.2026.1760632 (PMC13057567; doi:10.3389/fpls.2026.1760632)
Supplement: Supplementary Table 1 — Meteorological data recorded during Kharif 2024. [file Table1.docx]

**Supplementary Table 1: Meteorological data during *Kharif* 2024**

| **Jhansi** | | | | | | |
| --- | --- | --- | --- | --- | --- | --- |
| **S. No.** | **Month** | **Temperature** | | **Relative Humidity%** | | **Total Rainfall** |
|  |  | **Maximum** | **Minimum** | **I hour** | **II hour** | **(mm)** |
| 1 | June | 43.18 | 27.57 | 61.78 | 44.29 | 74.60 |
| 2 | July | 35.75 | 26.42 | 85.59 | 73.38 | 207.20 |
| 3 | August | 33.91 | 24.79 | 89.53 | NA | 288.80 |
| 4 | September | 33.29 | 32.54 | 91.89 | NA | 305.20 |
| 5 | October | 34.87 | 20.12 | 87.34 | 46.71 | 0.60 |
|  |  | 36.20 | 26.29 | 83.23 | 54.79 | 876.40 |
| **Amora** | | | | | | |
| 1 | June | 31.1 | 16.1 | 83.7 | 64.6 | 66.5 |
| 2 | July | 29.9 | 18.6 | 89.6 | 71.2 | 161 |
| 3 | August | 29.6 | 20 | 94.1 | 76.2 | 329.75 |
| 4 | September | 30.9 | 20.1 | 92.6 | 5 | 145.5 |
| 5 | October | 29 | 12.5 | 92.7 | 49 | 36.5 |
|  |  | 30.1 | 17.46 | 90.54 | 53.2 | 739.25 |
