# Supplementary material for: Variability and trait specific accessions for enhanced agronomic performance and nutritional traits in barnyard millet germplasm evaluated in diverse agro-ecologies in India
Source: Front Plant Sci. 2026 Mar 25;17:1760632. doi: 10.3389/fpls.2026.1760632 (PMC13057567; doi:10.3389/fpls.2026.1760632)
Supplement: Supplementary Table 2 — List of barnyard millet genotypes used in the study with their origin. [file Table2.docx]

**Supplementary table 2: Details of Barnyard millet genotypes used in the study**

| **S.N.** | **Genotypes** | **Origin** | **DOI** |
| --- | --- | --- | --- |
| 1 | IEC. 57 | India | <https://doi.org/10.18730/QND6> |
| 2 | IEC. 82 | India | <https://doi.org/10.18730/QNDZN> |
| 3 | IEC. 132 | India | <https://doi.org/10.18730/QNFH$> |
| 4 | IEC. 209 | India | <https://doi.org/10.18730/QNHYO> |
| 5 | IEC. 229 | India | <https://doi.org/10.18730/QNJJM> |
| 6 | IEC. 231 | India | <https://doi.org/10.18730/QNJMP> |
| 7 | IEC. 232 | India | <https://doi.org/10.18730/QNJNQ> |
| 8 | IEC. 239 | India | <https://doi.org/10.18730/QNJWY> |
| 9 | IEC. 265 | India | <https://doi.org/10.18730/QNKPK> |
| 10 | IEC. 269 | India | <https://doi.org/10.18730/QNKTQ> |
| 11 | IEC. 284 | India | <https://doi.org/10.18730/QNM91> |
| 12 | IEC. 346 | Syria | <https://doi.org/10.18730/QNP7T> |
| 13 | IEC. 379 | India | <https://doi.org/10.18730/QNQ8P> |
| 14 | IEC. 381 | India | <https://doi.org/10.18730/QNQAR> |
| 15 | IEC. 384 | India | <https://doi.org/10.18730/QNQDV> |
| 16 | IEC. 398 | India | <https://doi.org/10.18730/QNQV4> |
| 17 | IEC. 399 | India | <https://doi.org/10.18730/QNQW5> |
| 18 | IEC. 400 | India | <https://doi.org/10.18730/QNQX6> |
| 19 | IEC. 403 | Japan | <https://doi.org/10.18730/QNR09> |
| 20 | IEC. 436 | Japan | <https://doi.org/10.18730/QNS15> |
| 21 | IEC. 533 | Japan | <https://doi.org/10.18730/QNW2W> |
| 22 | IEC. 592 | India | <https://doi.org/10.18730/QNXXD> |
| 23 | IEC. 602 | India | <https://doi.org/10.18730/QNY7Q> |
| 24 | IEC. 631 | India | <https://doi.org/10.18730/QNZ4F> |
| 25 | IEC. 649 | India | <https://doi.org/10.18730/QNZP> ˜ |
| 26 | IEC. 749 | Unknown | <https://doi.org/10.18730/QP2TP> |
| 27 | IEC. 751 | Unknown | <https://doi.org/10.18730/QP2WR> |
| 28 | IEC. 331 | Russia | <https://doi.org/10.18730/QNNRB> |
| 29 | IEC. 332 | Russia | <https://doi.org/10.18730/QNNSC> |
| 30 | IEC. 344 | Russia | <https://doi.org/10.18730/QNP5R> |
| 31 | IEC. 348 | Malawi | <https://doi.org/10.18730/QNP9W> |
| 32 | IEC. 352 | Pakistan | <https://doi.org/10.18730/QNPD*> |
| 33 | IEC. 423 | Japan | <https://doi.org/10.18730/QNRMX> |
| 34 | IEC. 563 | Japan | <https://doi.org/10.18730/QNXON> |
| 35 | IEC. 624 | Cameroon | <https://doi.org/10.18730/QNYX8> |
| 36 | IEC. 654 | Pakistan | <https://doi.org/10.18730/QNZV1> |
| 37 | CO KV-2 | India |  |
| 38 | DHBM-93-3 | India |  |
| 39 | VL-Madira 207 | India |  |
