# Supplementary material for: Variability and trait specific accessions for enhanced agronomic performance and nutritional traits in barnyard millet germplasm evaluated in diverse agro-ecologies in India
Source: Front Plant Sci. 2026 Mar 25;17:1760632. doi: 10.3389/fpls.2026.1760632 (PMC13057567; doi:10.3389/fpls.2026.1760632)
Supplement: Supplementary file 3 [file Table3.docx]

**Table 3: Mean values of barnyard millet germplasm**

| **Genotypes** | **DTFF** | | **DTM** | | **FLL** | | **FLW** | | **PL** | | **PH** | |
| --- | --- | --- | --- | --- | --- | --- | --- | --- | --- | --- | --- | --- |
|  | **Jhansi** | **Almora** | **Jhansi** | **Almora** | **Jhansi** | **Almora** | **Jhansi** | **Almora** | **Jhansi** | **Almora** | **Jhansi** | **Almora** |
| IEC. 57 | 56.67 | 51.11 | 93.67 | 84.27 | 27.15 | 23.95 | 2.39 | 2.36 | 19.52 | 23.95 | 160.62 | 123.72 |
| IEC. 82 | 48.67 | 52.53 | 85.33 | 88.33 | 28.17 | 25.65 | 2.72 | 2.68 | 21.8 | 25.65 | 174.43 | 140.21 |
| IEC. 132 | 58.33 | 57.41 | 87.67 | 91.23 | 30.67 | 27.51 | 2.28 | 2.36 | 22.11 | 27.51 | 164.83 | 136.67 |
| IEC. 209 | 49.33 | 52.04 | 85 | 87.29 | 20.5 | 23.94 | 2.11 | 2.54 | 18.77 | 23.94 | 173.76 | 144.87 |
| IEC. 229 | 53.33 | 57.11 | 88.67 | 92.29 | 24.83 | 25.49 | 2.56 | 1.99 | 21.31 | 25.49 | 172.33 | 154.2 |
| IEC. 231 | 55.33 | 53.08 | 93.67 | 81.79 | 25.83 | 24.15 | 2.48 | 2.53 | 20.88 | 24.15 | 163.18 | 143.63 |
| IEC. 232 | 58.33 | 60.32 | 94.33 | 94.33 | 31.83 | 28.98 | 2.18 | 2.89 | 21.63 | 28.98 | 187.55 | 159.04 |
| IEC. 239 | 53.33 | 56.71 | 89.33 | 89.28 | 25.33 | 25.07 | 2.12 | 2.06 | 19.44 | 25.07 | 158.87 | 125.47 |
| IEC. 265 | 58.67 | 56.95 | 91.67 | 89.34 | 26.67 | 27.4 | 2.03 | 2.14 | 21.44 | 27.4 | 157.27 | 131.92 |
| IEC. 269 | 53.67 | 52.7 | 90.33 | 85.3 | 26 | 24.62 | 2.28 | 2.49 | 17.33 | 24.62 | 158.41 | 141.75 |
| IEC. 284 | 49.33 | 52.13 | 90 | 90.26 | 26.5 | 23.89 | 2.46 | 2.46 | 18.11 | 23.89 | 145.31 | 116.18 |
| IEC. 346 | 51.67 | 50.17 | 88.33 | 84.26 | 26.22 | 28.28 | 2.22 | 2.36 | 21.78 | 28.28 | 155.94 | 156.79 |
| IEC. 379 | 63.67 | 61.38 | 95.67 | 101.78 | 25.89 | 23.7 | 2.24 | 2.01 | 21 | 23.7 | 133.95 | 136.18 |
| IEC. 381 | 46.67 | 49.52 | 85.33 | 91.31 | 27.33 | 24.94 | 2.2 | 2.51 | 21.22 | 24.94 | 143.82 | 157.86 |
| IEC. 384 | 43.33 | 45.44 | 86 | 81.74 | 25.67 | 22.55 | 2.32 | 2.44 | 22.12 | 22.55 | 187.55 | 143.51 |
| IEC. 398 | 60.33 | 62.66 | 89.33 | 102.88 | 26.33 | 22.98 | 2.09 | 1.86 | 21 | 22.98 | 166.52 | 140.59 |
| IEC. 399 | 60.67 | 64.43 | 95.33 | 102.38 | 29 | 25.06 | 2.53 | 2.56 | 24.56 | 25.06 | 175.48 | 142.94 |
| IEC. 400 | 62.67 | 64.77 | 89 | 103.73 | 26.33 | 26.05 | 2.11 | 2.2 | 24.44 | 26.05 | 159.97 | 128.24 |
| IEC. 403 | 53 | 55.22 | 87.33 | 91.78 | 27.33 | 25.73 | 2.24 | 2.89 | 19 | 25.73 | 180.57 | 140.55 |
| IEC. 436 | 54.67 | 55.78 | 89.67 | 97.33 | 28.5 | 27.29 | 2.52 | 2.2 | 22 | 27.29 | 175.28 | 120.84 |
| IEC. 533 | 46.67 | 47.04 | 84.67 | 80.73 | 26.83 | 24.37 | 2.83 | 2.12 | 22.22 | 24.37 | 162.88 | 124.03 |
| IEC. 592 | 52.67 | 55.57 | 86.33 | 89.18 | 28.33 | 20.31 | 2.61 | 2.35 | 20.44 | 20.31 | 168.78 | 115.61 |
| IEC. 602 | 55 | 53.41 | 90.33 | 86.39 | 29 | 26.54 | 2.7 | 2.27 | 20.56 | 26.54 | 175.84 | 134.11 |
| IEC. 631 | 53 | 55.97 | 88 | 88.32 | 31.83 | 22.82 | 2.81 | 2.04 | 21.89 | 22.82 | 144.9 | 102.99 |
| IEC. 649 | 42.33 | 44.94 | 88.33 | 86.98 | 27.83 | 27.53 | 2.36 | 1.78 | 19.89 | 27.53 | 159.5 | 128.46 |
| IEC. 749 | 51.67 | 52.1 | 88.67 | 90.22 | 26.17 | 22.76 | 2.69 | 1.83 | 18.67 | 22.76 | 144.56 | 111.63 |
| IEC. 751 | 58.67 | 61.26 | 93.33 | 102.68 | 25.33 | 22.26 | 2.48 | 1.82 | 20.56 | 22.26 | 180.15 | 139.88 |
| IEC. 331 | 53.67 | 52.74 | 91 | 89.83 | 28.33 | 25.82 | 2.44 | 1.99 | 21.78 | 25.82 | 156.97 | 137.51 |
| IEC. 332 | 55.33 | 57.09 | 92.33 | 82.33 | 26.17 | 31.12 | 2.24 | 2.59 | 21.89 | 31.12 | 183.79 | 155.55 |
| IEC. 344 | 47.33 | 46.58 | 88.67 | 82.34 | 30 | 29.31 | 2.2 | 2.48 | 19.33 | 29.31 | 159.78 | 144.53 |
| IEC. 348 | 53.33 | 56.79 | 90 | 96.79 | 24.5 | 24.62 | 2.39 | 2.08 | 19 | 24.62 | 138.92 | 123.08 |
| IEC. 352 | 62.67 | 64.63 | 87 | 103.7 | 25.67 | 27.66 | 2.48 | 2.07 | 22.22 | 27.66 | 155.14 | 125.22 |
| IEC. 423 | 61.33 | 60.55 | 89.33 | 100.67 | 24.5 | 20.59 | 2.49 | 1.79 | 21.22 | 20.59 | 151.34 | 133.41 |
| IEC. 563 | 57.67 | 55.5 | 89.67 | 93.73 | 25.17 | 24.48 | 2.27 | 1.92 | 19.56 | 24.48 | 152 | 134.57 |
| IEC. 624 | 58.33 | 56.1 | 88.33 | 84.4 | 29.5 | 32.33 | 2.67 | 2.44 | 21.79 | 32.33 | 171.11 | 159.15 |
| IEC. 654 | 54.67 | 52.02 | 91.33 | 88.27 | 28.09 | 26.93 | 2.32 | 2.46 | 21.89 | 26.93 | 162.22 | 124.68 |
| CO KV-2 | 58 | 56.6 | 91.33 | 97.38 | 24.28 | 28.6 | 2.53 | 1.81 | 24.41 | 28.6 | 143.72 | 110.19 |
| DHBM-93-3 | 56.67 | 57.55 | 92.33 | 104.23 | 28.83 | 25.22 | 2.04 | 2.64 | 20.56 | 25.22 | 170.57 | 121.16 |
| VL-Madira 207 | 58.33 | 56.55 | 88.33 | 95.78 | 24.33 | 20.38 | 2.31 | 2.45 | 21.22 | 20.38 | 147.43 | 143.31 |
| **Minimum** | 42.33 | 44.94 | 84.67 | 80.73 | 20.5 | 20.31 | 2.03 | 1.78 | 17.33 | 20.31 | 133.95 | 102.99 |
| **Maximum** | 63.67 | 64.77 | 94.67 | 104.23 | 31.83 | 32.33 | 2.83 | 2.89 | 24.56 | 32.33 | 187.55 | 159.15 |
| **CD** | 1.97 | 1.695 | 5.436 | 2.353 | 2.876 | 0.313 | 0.48 | 0.313 | 2.377 | 2.33 | 12.38 | 3.73 |
| **SE(m)** | 0.698 | 0.6 | 1.926 | 0.834 | 1.019 | 0.111 | 0.17 | 0.111 | 0.842 | 0.82 | 4.38 | 1.32 |
| **SE(d)** | 0.987 | 0.849 | 2.724 | 1.179 | 1.441 | 0.157 | 0.241 | 0.157 | 1.191 | 1.16 | 6.2 | 1.87 |
| **C.V** | 2.215 | 1.883 | 3.723 | 1.576 | 6.568 | 8.469 | 12.359 | 8.469 | 6.952 | 5.63 | 4.68 | 1.7 |

| **Genotypes** | **GY** | | **Protein** | | **Lipid** | | **Carbohydrate** | | **Crude fibre** | | **Ash** | |
| --- | --- | --- | --- | --- | --- | --- | --- | --- | --- | --- | --- | --- |
|  | **Jhansi** | **Almora** | **Jhansi** | **Almora** | **Jhansi** | **Almora** | **Jhansi** | **Almora** | **Jhansi** | **Almora** | **Jhansi** | **Almora** |
| IEC. 57 | 15.52 | 16.485 | 7.02 | 7.16 | 6.46 | 7.06 | 64.85 | 61.97 | 6.5 | 6.17 | 2.21 | 2.66 |
| IEC. 82 | 19.58 | 20.635 | 6.92 | 7.38 | 6.78 | 7.1 | 65 | 70.19 | 6.43 | 5.35 | 2.42 | 2.32 |
| IEC. 132 | 15.53 | 18.095 | 5.85 | 6.61 | 5.77 | 5.89 | 61.5 | 64.14 | 5.7 | 6.3 | 2.5 | 2.84 |
| IEC. 209 | 18.05 | 19.4 | 6.69 | 6.29 | 6.12 | 5.52 | 63.2 | 59.18 | 6.55 | 7.01 | 2.4 | 2.98 |
| IEC. 229 | 15.78 | 14.63 | 6.73 | 6.95 | 6.59 | 5.91 | 61.15 | 67.43 | 6.35 | 5.88 | 2.5 | 3.2 |
| IEC. 231 | 13.94 | 15.24 | 6.26 | 5.97 | 6.51 | 6.91 | 68.2 | 64.03 | 5.85 | 5.63 | 2.35 | 2.66 |
| IEC. 232 | 20.04 | 22.335 | 7.28 | 6.67 | 6.04 | 5.33 | 66.5 | 63.68 | 6.6 | 6.77 | 2.35 | 2.12 |
| IEC. 239 | 13.42 | 12.69 | 7.55 | 7.22 | 6.38 | 6.14 | 72.5 | 75.52 | 6.05 | 6.33 | 2.5 | 2.41 |
| IEC. 265 | 14.43 | 12.63 | 7.11 | 6.52 | 6.7 | 6.07 | 66.15 | 68.16 | 5.15 | 6.02 | 2.05 | 2.24 |
| IEC. 269 | 13.98 | 15.685 | 6.12 | 7.99 | 6.77 | 7.02 | 66.1 | 63.72 | 5.02 | 5.7 | 2.45 | 2.83 |
| IEC. 284 | 14.87 | 12.585 | 5.23 | 6.01 | 6.39 | 5.44 | 71.75 | 63.35 | 6.45 | 6.25 | 2.25 | 2.61 |
| IEC. 346 | 15.42 | 14.605 | 7.15 | 7.12 | 7.16 | 7.25 | 67 | 60.25 | 6.4 | 6.66 | 2.35 | 2.32 |
| IEC. 379 | 9.31 | 12.9 | 6.02 | 7.24 | 7.62 | 8.08 | 74.65 | 72.03 | 5.8 | 6.45 | 2.8 | 2.12 |
| IEC. 381 | 16.21 | 18.575 | 5.92 | 6.7 | 7.99 | 7.94 | 56.1 | 55.53 | 6.3 | 6.38 | 2.8 | 2.94 |
| IEC. 384 | 13.19 | 10.255 | 6.45 | 7.15 | 6.17 | 5.84 | 62.15 | 64.98 | 5.6 | 4.9 | 2.65 | 3.34 |
| IEC. 398 | 9.46 | 8.6 | 7.05 | 7.8 | 6.18 | 5.95 | 67.8 | 69.2 | 5.55 | 4.86 | 2.3 | 2.61 |
| IEC. 399 | 17.51 | 18.37 | 6.75 | 6.31 | 6.94 | 7.06 | 55.1 | 63.75 | 6.5 | 6.58 | 2.45 | 2.37 |
| IEC. 400 | 14.82 | 13.185 | 6.1 | 6.51 | 8.05 | 7.42 | 57.35 | 57.96 | 6.1 | 6.44 | 2.55 | 2.75 |
| IEC. 403 | 18.93 | 17.215 | 6.25 | 7.88 | 6.53 | 7.6 | 62.75 | 66.24 | 6.3 | 5.88 | 2.65 | 2.52 |
| IEC. 436 | 17.08 | 13.015 | 6.9 | 6.82 | 6.66 | 8 | 66.15 | 69.25 | 6.2 | 6.53 | 2.4 | 2.82 |
| IEC. 533 | 15.1 | 14.4 | 7.1 | 6.11 | 6.79 | 5.99 | 61.8 | 64.09 | 5.45 | 5.59 | 2.35 | 3.23 |
| IEC. 592 | 16.34 | 14.265 | 6.45 | 7.55 | 7.22 | 7.01 | 61.05 | 66.37 | 6.35 | 6.73 | 2.05 | 2.43 |
| IEC. 602 | 17.8 | 15.305 | 6.85 | 6.92 | 7.35 | 6.07 | 71.7 | 73.83 | 5.85 | 7.1 | 2.05 | 2.66 |
| IEC. 631 | 12.5 | 10.485 | 5.75 | 7.11 | 6.28 | 6.35 | 66.1 | 65.83 | 6.2 | 5.8 | 2.45 | 2.43 |
| IEC. 649 | 14.28 | 13.1 | 6.65 | 7.04 | 6.47 | 8.32 | 63.1 | 63.21 | 5.75 | 5.89 | 2.45 | 2.68 |
| IEC. 749 | 12.07 | 11.33 | 5.85 | 5.36 | 6.72 | 6.61 | 64.25 | 70.01 | 6.65 | 7.09 | 2.2 | 2.08 |
| IEC. 751 | 12.97 | 14.71 | 6.25 | 6.99 | 5.81 | 5.58 | 64.25 | 61.28 | 6.35 | 7.09 | 2.4 | 2.66 |
| IEC. 331 | 13.85 | 11.855 | 5.95 | 6.25 | 6.85 | 6.96 | 52.65 | 60.57 | 5.35 | 5.93 | 2.4 | 2.11 |
| IEC. 332 | 14.03 | 15.82 | 6.97 | 8.14 | 7.01 | 6.41 | 64.1 | 55.08 | 6.25 | 6.62 | 2.55 | 2.57 |
| IEC. 344 | 18.85 | 17.49 | 6.25 | 6.32 | 6.94 | 7.49 | 57.2 | 56.27 | 5.5 | 6.79 | 2.55 | 2.75 |
| IEC. 348 | 12.06 | 10.72 | 6.15 | 5.98 | 6.4 | 6.2 | 63.35 | 62.93 | 5.55 | 5.43 | 2 | 2.28 |
| IEC. 352 | 13.36 | 10.16 | 7 | 7.25 | 5.43 | 6.07 | 65.75 | 64.46 | 6.1 | 6.55 | 2.05 | 2.73 |
| IEC. 423 | 13.03 | 14.29 | 6.3 | 7.33 | 7.01 | 7.93 | 72.1 | 72.77 | 6.1 | 6.39 | 2.15 | 2.49 |
| IEC. 563 | 12.86 | 11.91 | 6.25 | 5.93 | 6.41 | 6.13 | 59.15 | 57.25 | 6.3 | 5.72 | 2.6 | 2.56 |
| IEC. 624 | 15.93 | 17.905 | 6.75 | 6.87 | 6.44 | 6.13 | 66.5 | 72.91 | 6.15 | 6.63 | 2.6 | 2.42 |
| IEC. 654 | 14.88 | 11.345 | 5.9 | 6.38 | 6.23 | 5.99 | 63.8 | 66.82 | 6.2 | 7.2 | 2.4 | 3.11 |
| CO KV-2 | 12.02 | 10.125 | 6.75 | 5.82 | 5.42 | 5.25 | 55.55 | 62.33 | 6.4 | 6.7 | 2.6 | 3.06 |
| DHBM-93-3 | 15.83 | 18.78 | 6.15 | 6.58 | 6.68 | 7.94 | 58.84 | 64.11 | 6.6 | 5.59 | 2.45 | 2.28 |
| VL-Madira 207 | 16.49 | 14.62 | 6.95 | 7.21 | 6.26 | 6.8 | 66.05 | 73.71 | 6.2 | 7.05 | 2.5 | 2.75 |
| **Minimum** | 9.31 | 8.6 | 5.23 | 5.36 | 5.42 | 5.25 | 52.65 | 55.08 | 5.02 | 4.86 | 2 | 2.08 |
| **Maximum** | 20.04 | 22.33 | 7.55 | 8.14 | 8.05 | 8.32 | 74.65 | 75.52 | 6.65 | 7.2 | 2.8 | 3.34 |
| **CD** | 2.489 | 1.453 | 0.79 | 0.796 | 1.154 | 0.692 | 3.524 | 3.652 | 0.383 | 0.463 | 0.356 | 0.315 |
| **SE(m)** | 0.882 | 0.515 | 0.28 | 0.282 | 0.409 | 0.245 | 1.249 | 1.294 | 0.136 | 0.164 | 0.126 | 0.112 |
| **SE(d)** | 1.247 | 0.728 | 0.39 | 0.399 | 0.578 | 0.347 | 1.766 | 1.83 | 0.192 | 0.232 | 0.179 | 0.158 |
| **C.V** | 10.249 | 6.147 | 7.45 | 7.178 | 10.694 | 6.43 | 3.383 | 3.449 | 3.873 | 4.546 | 9.099 | 7.403 |

| **Genotypes** | **TSS** | | **TAA** | | **TPC** | | **Fe** | | **Zn** | |
| --- | --- | --- | --- | --- | --- | --- | --- | --- | --- | --- |
|  | **Jhansi** | **Almora** | **Jhansi** | **Almora** | **Jhansi** | **Almora** | **Jhansi** | **Almora** | **Jhansi** | **Almora** |
| IEC. 57 | 23.15 | 24.48 | 25.63 | 26.4 | 3.37 | 4.11 | 39.2 | 42.24 | 25.1 | 26.62 |
| IEC. 82 | 21.31 | 21.96 | 27.34 | 29.36 | 2.9 | 3.69 | 50.89 | 53.28 | 26.07 | 27.47 |
| IEC. 132 | 20.66 | 17.66 | 32.65 | 32.29 | 2.83 | 2.54 | 57.04 | 51.95 | 26.15 | 29.7 |
| IEC. 209 | 20.45 | 22.42 | 26.64 | 25.03 | 3.08 | 3.89 | 47.3 | 45.95 | 22.9 | 23.91 |
| IEC. 229 | 20.34 | 21 | 30.07 | 26.12 | 3.29 | 2.86 | 40.91 | 46.08 | 24.78 | 26.47 |
| IEC. 231 | 21.43 | 22.68 | 26.81 | 33.78 | 3 | 3.89 | 43.32 | 42.67 | 26.44 | 28.64 |
| IEC. 232 | 19.93 | 21.32 | 31.28 | 34.55 | 3.49 | 3.69 | 59.15 | 57.57 | 27.44 | 24.88 |
| IEC. 239 | 23.51 | 24.71 | 27.96 | 24.91 | 3 | 3.85 | 43.46 | 47.14 | 30.18 | 27.55 |
| IEC. 265 | 21.99 | 18.94 | 33.37 | 34.67 | 4.74 | 4.31 | 36.85 | 33.49 | 38.42 | 35.54 |
| IEC. 269 | 25.72 | 24.47 | 25.77 | 23.57 | 3.1 | 2.59 | 37.86 | 36.07 | 29.96 | 32.25 |
| IEC. 284 | 22.6 | 22.88 | 18.87 | 22.21 | 3.71 | 4.23 | 65.46 | 63.78 | 31.31 | 34.66 |
| IEC. 346 | 22.34 | 24.83 | 26.06 | 29.87 | 3.19 | 4.09 | 64.9 | 66.53 | 34.26 | 34.8 |
| IEC. 379 | 21.97 | 25 | 26.13 | 28.62 | 4.96 | 5.5 | 54.27 | 58.78 | 35.76 | 37.41 |
| IEC. 381 | 23.34 | 23.52 | 22.78 | 24.31 | 3.11 | 2.31 | 54.62 | 55.7 | 29.52 | 27.41 |
| IEC. 384 | 25.09 | 26.45 | 31.42 | 27.65 | 2.78 | 2.57 | 45.33 | 48.07 | 27.65 | 26.79 |
| IEC. 398 | 24.09 | 28.75 | 32.12 | 33.94 | 3.75 | 4.36 | 50.56 | 48.28 | 17.76 | 18.56 |
| IEC. 399 | 24.95 | 23.99 | 26.36 | 24.3 | 3.17 | 3.22 | 59.92 | 60.8 | 18.38 | 22.35 |
| IEC. 400 | 20.86 | 20.51 | 30.79 | 27.63 | 2.83 | 4.36 | 63 | 63.98 | 20.42 | 24.3 |
| IEC. 403 | 26.01 | 26.51 | 29.29 | 31.96 | 4.8 | 5.39 | 47.14 | 50.55 | 15.47 | 16 |
| IEC. 436 | 24.14 | 29.03 | 15.65 | 19.92 | 2.95 | 2.94 | 51.13 | 53.91 | 18.63 | 16.78 |
| IEC. 533 | 26.9 | 26.24 | 28.74 | 24.66 | 2.75 | 2.8 | 40.52 | 44.5 | 14.83 | 14.46 |
| IEC. 592 | 24.46 | 25.24 | 31.39 | 30.76 | 2.75 | 3.84 | 49.97 | 53.09 | 17.7 | 15.92 |
| IEC. 602 | 22.74 | 22.31 | 26.8 | 24.78 | 2.84 | 3.19 | 51.52 | 46.32 | 18.55 | 17.96 |
| IEC. 631 | 22.07 | 21.31 | 32.29 | 34.58 | 3.5 | 3.74 | 58.42 | 62.94 | 19.33 | 22.37 |
| IEC. 649 | 20.18 | 23.86 | 29.24 | 31.84 | 3.07 | 3.74 | 46.77 | 49.47 | 16.33 | 17.47 |
| IEC. 749 | 21.5 | 19.42 | 22.02 | 24.1 | 2.81 | 3.6 | 57.19 | 57.54 | 19.75 | 16.95 |
| IEC. 751 | 20.55 | 21.48 | 21.56 | 24.47 | 2.83 | 3.13 | 58.8 | 59.42 | 19.13 | 17.69 |
| IEC. 331 | 23.15 | 25.16 | 26.59 | 24.06 | 2.67 | 3.01 | 46.07 | 48.35 | 16.85 | 17.69 |
| IEC. 332 | 25.25 | 27.06 | 28.72 | 31.45 | 2.76 | 3.74 | 54.09 | 57.32 | 16.53 | 21.75 |
| IEC. 344 | 21.8 | 21.38 | 25.98 | 28.96 | 2.63 | 2.39 | 61.04 | 63.86 | 17.31 | 15.1 |
| IEC. 348 | 26.45 | 28.05 | 23.89 | 25.52 | 3.6 | 4.11 | 51.59 | 44.63 | 19.17 | 21.81 |
| IEC. 352 | 21.75 | 24.32 | 23.05 | 21.31 | 2.56 | 3.11 | 42.75 | 53.48 | 22.34 | 25.32 |
| IEC. 423 | 19.3 | 22.89 | 26.16 | 27.2 | 2.66 | 2.49 | 42.07 | 47.49 | 19.24 | 22.77 |
| IEC. 563 | 21.4 | 20.07 | 19.22 | 23.17 | 2.54 | 3.09 | 42.08 | 37.68 | 18.17 | 15.62 |
| IEC. 624 | 25.5 | 25.75 | 23.14 | 27.11 | 2.85 | 2.52 | 40.4 | 43.92 | 26.19 | 27.97 |
| IEC. 654 | 23 | 26.17 | 26.08 | 23.91 | 3.83 | 2.94 | 44.69 | 42.28 | 23.58 | 25.95 |
| CO KV-2 | 24.75 | 23.62 | 22.75 | 28.29 | 3.95 | 4.24 | 51.31 | 51.94 | 25.81 | 23.11 |
| DHBM-93-3 | 26.5 | 26.66 | 27.45 | 33.45 | 3.38 | 3.21 | 43.63 | 45.7 | 20.56 | 26.14 |
| VL-Madira 207 | 25.25 | 24.35 | 25.94 | 27.06 | 3.27 | 3.34 | 35.93 | 39.51 | 20.25 | 25.25 |
| **Minimum** | 19.3 | 17.66 | 15.65 | 19.92 | 2.54 | 2.31 | 35.93 | 33.49 | 14.83 | 14.46 |
| **Maximum** | 26.9 | 29.03 | 33.37 | 34.67 | 4.96 | 5.5 | 65.46 | 66.53 | 38.42 | 37.41 |
| **CD** | 2.673 | 2.098 | 3.469 | 2.499 | 0.34 | 0.65 | 5.714 | 5.285 | 0.564 | 1.763 |
| **SE(m)** | 0.947 | 0.743 | 1.229 | 0.886 | 0.12 | 0.23 | 2.025 | 1.873 | 0.2 | 0.625 |
| **SE(d)** | 1.34 | 1.051 | 1.738 | 1.252 | 0.17 | 0.32 | 2.863 | 2.648 | 0.283 | 0.884 |
| **C.V** | 7.139 | 5.42 | 8 | 5.551 | 6.62 | 11.3 | 7.082 | 6.401 | 1.503 | 4.522 |

| **Genotypes** | **Ca** | | | | **Mg** | | | |
| --- | --- | --- | --- | --- | --- | --- | --- | --- |
|  | **Jhansi** | | **Almora** | | **Jhansi** | | **Almora** | |
| IEC. 57 | 34.73 | | 38.75 | | 70.5 | | 67.04 | |
| IEC. 82 | 25.55 | | 26.31 | | 61.5 | | 61.63 | |
| IEC. 132 | 35.6 | | 37.14 | | 68.65 | | 69.4 | |
| IEC. 209 | 40.54 | | 43.16 | | 74.55 | | 74.17 | |
| IEC. 229 | 29.8 | | 25.66 | | 74.75 | | 73.81 | |
| IEC. 231 | 35.6 | | 34.66 | | 59.3 | | 63.27 | |
| IEC. 232 | 27.6 | | 30.7 | | 67.9 | | 63.85 | |
| IEC. 239 | 32.1 | | 37.29 | | 66.45 | | 59.46 | |
| IEC. 265 | 40.05 | | 34.34 | | 68.55 | | 73.31 | |
| IEC. 269 | 24.8 | | 29.33 | | 61.75 | | 63.74 | |
| IEC. 284 | 25.6 | | 27.29 | | 67.5 | | 68.72 | |
| IEC. 346 | 26.1 | | 34.38 | | 70.5 | | 73.39 | |
| IEC. 379 | 21.8 | | 25.73 | | 73.7 | | 69.22 | |
| IEC. 381 | 23.5 | | 27.45 | | 68.35 | | 71.27 | |
| IEC. 384 | 25 | | 24.82 | | 47.85 | | 54.42 | |
| IEC. 398 | 30.85 | | 25.62 | | 57.15 | | 59.33 | |
| IEC. 399 | 21.4 | | 24.92 | | 56.5 | | 60.39 | |
| IEC. 400 | 25 | | 21.95 | | 62.5 | | 64.34 | |
| IEC. 403 | 35.8 | | 36.07 | | 59.8 | | 54.78 | |
| IEC. 436 | 22.5 | | 20.58 | | 70 | | 74.39 | |
| IEC. 533 | 34.5 | | 34.12 | | 62.5 | | 64.1 | |
| IEC. 592 | 37.15 | | 34.76 | | 56.25 | | 64.86 | |
| IEC. 602 | 27.05 | | 25.96 | | 62.5 | | 57.4 | |
| IEC. 631 | 31.5 | | 34.36 | | 44.7 | | 43.52 | |
| IEC. 649 | | 25.4 | | 25.86 | | 43 | | 53.85 |
| IEC. 749 | | 25.95 | | 24.81 | | 54.75 | | 57.18 |
| IEC. 751 | | 21.2 | | 24.95 | | 66.5 | | 72.67 |
| IEC. 331 | | 25.5 | | 30.34 | | 45.9 | | 57.48 |
| IEC. 332 | | 33.7 | | 33.16 | | 52.3 | | 53.83 |
| IEC. 344 | | 25.3 | | 28.61 | | 65.3 | | 66.43 |
| IEC. 348 | | 34.35 | | 30.31 | | 66.19 | | 68.86 |
| IEC. 352 | | 24 | | 27.92 | | 55.5 | | 62.02 |
| IEC. 423 | | 31.5 | | 33.16 | | 63.5 | | 63.92 |
| IEC. 563 | | 23 | | 25.5 | | 74 | | 69.31 |
| IEC. 624 | | 25 | | 30.57 | | 69 | | 74.8 |
| IEC. 654 | | 33 | | 35.99 | | 66.5 | | 66.19 |
| CO KV-2 | | 30.5 | | 35.78 | | 69.8 | | 76.43 |
| DHBM-93-3 | | 24.5 | | 30.41 | | 65.5 | | 70.4 |
| VL-Madira 207 | | 27.85 | | 27.96 | | 56.5 | | 63.89 |
| **Minimum** | | 21.2 | | 20.58 | | 43 | | 43.52 |
| **Maximum** | | 40.54 | | 43.16 | | 74.75 | | 76.43 |
| **CD** | | 4.293 | | 2.817 | | 4.045 | | 2.388 |
| **SE(m)** | | 1.521 | | 0.998 | | 1.433 | | 0.846 |
| **SE(d)** | | 2.151 | | 1.412 | | 2.027 | | 1.197 |
| **C.V** | | 9.134 | | 5.711 | | 3.955 | | 2.262 |

DTFF = Days to 50% flowering (number of days), DTM = Days to maturity (number of days), FLL = Flag leaf length (cm), FLW = Flag leaf width (cm), PL = Panicle length (cm), PH = Plant height (cm), GY = Grain yield (g/plant), Protein (%), Lipid (%), Carbohydrate (%), Crude fibre (%), Ash (%), TSS = Total soluble sugars (mg/100mg), TAA = Total antioxidant activity (mM trolox equivalent/g dw), TPC = Total polyphenol content (mg GAE/100g), Fe = Iron (mg/1000g), Zn = Zinc (mg/1000g), Ca = Calcium (mg/100g), Mg = Magnesium (mg/100g)
